# Arginine starvation kills tumor cells through aspartate exhaustion and mitochondrial dysfunction

**DOI:** 10.1038/s42003-018-0178-4

**Published:** 2018-10-26

**Authors:** Chun-Ting Cheng, Yue Qi, Yi-Chang Wang, Kevin K. Chi, Yiyin Chung, Ching Ouyang, Yun-Ru Chen, Myung Eun Oh, Xiangpeng Sheng, Yulong Tang, Yun-Ru Liu, H. Helen Lin, Ching-Ying Kuo, Dustin Schones, Christina M. Vidal, Jenny C.-Y. Chu, Hung-Jung Wang, Yu-Han Chen, Kyle M. Miller, Peiguo Chu, Yun Yen, Lei Jiang, Hsing-Jien Kung, David K. Ann

**Affiliations:** 1 Department of Diabetes Complications and Metabolism, Diabetes and Metabolism Research Institute, City of Hope, Duarte, CA 91010 USA; 2 Irell and Manella Graduate School of Biological Sciences, City of Hope, Duarte, CA 91010 USA; 3 Department of Information Sciences, City of Hope, Duarte, CA 91010 USA; 4 Department of Molecular and Cellular Endocrinology, City of Hope, Duarte, CA 91010 USA; 5 Office of Human Research, Center for Cancer Research, Taipei Medical University, Taipei City, Taiwan; 6 Institute for Translational Medicine, Taipei Medical University, Taipei City, Taiwan; 7 Institute of Biotechnology and Pharmaceutical Research, National Health Research Institutes, Miaoli County, Taiwan; 8 Institute of Molecular and Genomic Medicine, National Health Research Institutes, Miaoli County, Taiwan; 9 UC Irvine Diabetes Center, University of California at Irvine, Irvine, CA 92697 USA; 10 Department of Molecular Biosciences, Institute for Cellular and Molecular Biology, The University of Texas at Austin, Austin, TX 78712 USA; 11 Shared Pathology Core, City of Hope, Duarte, CA 91010 USA; 12 Department of Biochemistry and Molecular Medicine, University of California at Davis Comprehensive Cancer Center, Sacramento, CA 95817 USA

## Abstract

Defective arginine synthesis, due to the silencing of *argininosuccinate synthase 1* (ASS1), is a common metabolic vulnerability in cancer, known as arginine auxotrophy. Understanding how arginine depletion kills arginine-auxotrophic cancer cells will facilitate the development of anti-cancer therapeutic strategies. Here we show that depletion of extracellular arginine in arginine-auxotrophic cancer cells causes mitochondrial distress and transcriptional reprogramming. Mechanistically, arginine starvation induces asparagine synthetase (ASNS), depleting these cancer cells of aspartate, and disrupting their malate-aspartate shuttle. Supplementation of aspartate, depletion of mitochondria, and knockdown of ASNS all protect the arginine-starved cells, establishing the causal effects of aspartate depletion and mitochondrial dysfunction on the arginine starvation-induced cell death. Furthermore, dietary arginine restriction reduced tumor growth in a xenograft model of ASS1-deficient breast cancer. Our data challenge the view that ASNS promotes homeostasis, arguing instead that ASNS-induced aspartate depletion promotes cytotoxicity, which can be exploited for anti-cancer therapies.

## Abstract (web summary)

Chun-Ting Cheng et al. demonstrate that arginine starvation kills arginine-auxotrophic cancer cells by depleting them of aspartate through asparagine synthetase (ASNS) and disrupting their mitochondrial metabolism. This study presents ASNS-induced aspartate depletion as an anti-cancer therapeutic strategy.

## Introduction

Due to metabolic shifts, many cancer cells come to depend on the presence of exogenous amino acids^1–7^. For instance, in non-cancerous cells arginine is synthesized in cells from citrulline via argininosuccinate synthase 1 (ASS1) and argininosuccinate lyase in the urea cycle^8^, and metabolized by arginase 1 to produce urea and ornithine. Ornithine is a precursor for the biosynthesis of polyamines and proline, which are required for a wide variety of cellular functions^9,10^. Downregulation of urea cycle components, which shunts metabolites away from arginine synthesis and toward pyrimidine biosynthesis to support cell proliferation, is frequently found as part of cancer metabolic reprograming^11^. Therefore, extrinsic (dietary) arginine, which is nonessential in non-cancerous human cells, becomes critical to the survival of cancer cells, a condition known as arginine auxotrophy. A defect in arginine synthesis is one of the most common, yet under-recognized, metabolic vulnerabilities in cancer^12^.

Mitochondrial function is often altered by cancer cells as a metabolic adaption to high energy demands^13^. An emerging concept is that mitochondria also function as signaling organelles^14,15^. Three notable mitochondria-dependent signaling mechanisms involve the production of ROS, acetyl-CoA, and α-ketoglutarate. Excess ROS damage cellular macromolecules, including DNA, resulting in genome instability^16^. The levels of acetyl-CoA and α-ketoglutarate regulate acetylation and methylation of histone proteins, respectively^17–19^, which alters DNA accessibility and function, including transcription. We and others have shown that arginine starvation damages mitochondria, which results in elevated accumulation of excess ROS and subsequent genome instability, eventually leading to a novel form of arginine auxotrophic cell death called chromatophagy^3,6,13,20–26^.

In this report, we show that mitochondrial dysregulation, including impaired respiration and transcriptional downregulation, links arginine starvation and cell death. We also uncover an important role for endoplasmic reticulum (ER) proteostasis perturbation, referred as ER stress^27^, which causes ATF4-dependent ASNS induction and aspartate depletion in arginine-starved cells. Thus, the fate of arginine-starved cells is impacted by mitochondrial dysregulation and the availability of intracellular aspartate, which regulates NADH and nucleotide production. In support of arginine restriction as a therapeutic strategy, we find that feeding an arginine restricted diet suppresses the growth of arginine auxotrophic tumors in a xenograft model. Altogether, this study provides novel insights into the mechanisms underlying the vulnerability of arginine auxotrophic cancer cells to arginine starvation.

## Results

### Impact of arginine starvation on TCA cycle and glycolysis

Previously, we showed that low ASS1 abundance predicts poor breast cancer survival^6^. To characterize ASS1 abundance in human cancers, we examined *ASS1* expression using The Cancer Genome Atlas (TCGA) pan-cancer data^28^. *ASS1* expression was downregulated in multiple human cancer types (12 of 14 investigated cancer types; 10 with statistical significance) (Supplementary Fig. 1), suggesting that arginine auxotrophy is a common phenomenon in multiple cancer types.

**Fig. 1:**
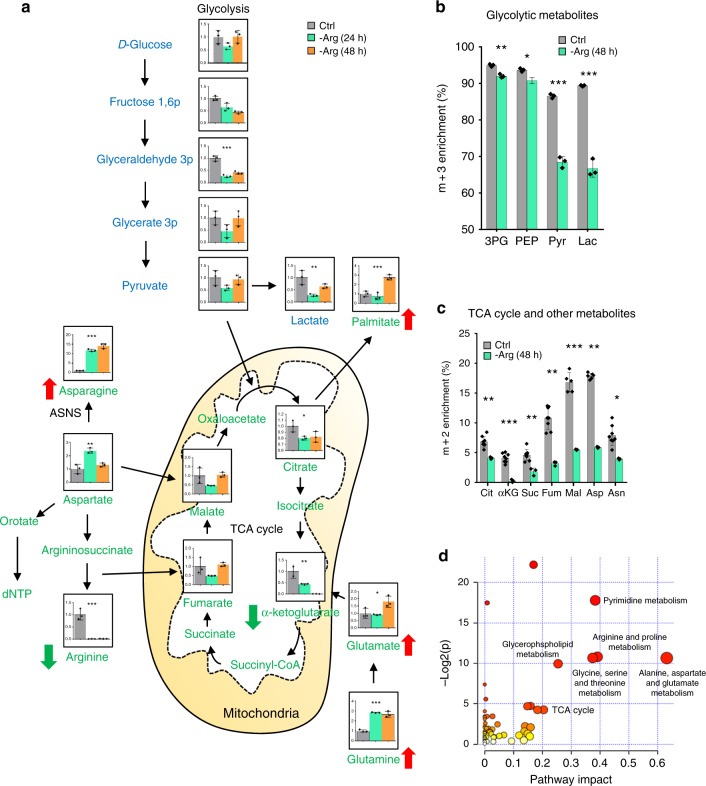
Metabolomics analysis of MDA-MB-231 cells upon arginine starvation. **a** Selected glycolysis (blue text) and TCA cycle (green text) intermediates, as well as amino acids, were quantified using mass spectrometry in MDA-MB-231 cells. Equal numbers of cells (about 70% confluency) were harvested after culture in arginine-replenished (Ctrl) or arginine-starved (-Arg) medium for 24 and 48 h. Data are shown as mean ± S.D.; *n* = 3; *p *<* 0.05; **p *<* 0.01; ***p *<* 0.001. **b**, **c** Arginine starvation (48 h) suppresses flux from [U-^13^C]glucose to glycolysis (**b**) and TCA cycle (**c**) in MDA-MB-231. Equal numbers of cells (about 70% confluency) were harvested after culture in arginine-replenished (Ctrl) or arginine-starved (-Arg) medium for 24 and 48 h. Data are shown as mean ± S.D.; *n* ≥ 3; *p *<* 0.05; **p *<* 0.01; ***p *<* 0.001. Experiments were repeated at least three times. **d** Overview of pathway analysis. The scatterplot represents the pathway impact value and *p*-value from pathway topology analysis of the differentially expressed metabolites from MDA-MB-231 cells cultured in full and arginine-starved (48 h) medium. The size and color of each node is based on its pathway impact value and *p*-value, respectively. Pathways with statistical significance (*p* < 0.05) are shown in red.

We analyzed metabolic footprint resulting from arginine starvation by exposure of ASS1-deficient MDA-MB-231 breast cancer cells to arginine free medium. One hundred and sixteen metabolites were detected and quantified with accurate mass measurements and retention times using TraceFinder 3.3. First, we confirmed that arginine is the most notably decreased amino acid (by approximate 50-fold) upon arginine starvation (Fig. 1a, Supplementary Fig. 2A). Next, the effect of arginine starvation on glycolysis and TCA cycle were further analyzed in MDA-MB-231 cells by ^13^C-labeling techniques using [U-^13^C] glucose as tracers. ^13^C-labeling analysis of intracellular metabolites demonstrated that glucose was metabolized mainly via glycolysis (Fig. 1b), and that glycolysis flux was reduced by arginine starvation (~20% reduction in m + 3-labeled pyruvate and lactate from [U-^13^C] glucose in arginine-starved MDA-MB-231 cells) (Fig. 1b). In contrast, relatively low percentage of m + 2-labeled TCA cycle intermediates and amino acids of aspartate and asparagine from [U-^13^C] glucose (~10%) (Fig. 1c). Notably, the TCA cycle fluxes from glucose into the TCA cycle (m + 2) were reduced by arginine starvation (48 h).

Both heatmap and metabolic impact analyses segregated the samples by metabolic pathway, with replicates tightly grouped (Fig. 1d, Supplementary Fig. 2A). To illustrate the effects of transferring the MDA-MB-231 cells from full medium to arginine-starved medium (24 h and 48 h), we selected 70 significantly altered metabolites and performed pathway analysis based on KEGG pathway maps (Release 82.1)^29^. Our metabolic impact and pathway enrichment analyses showed that amino acid metabolism, pyrimidine metabolism, glycerophospholipid metabolism and the TCA cycle were the metabolic pathways most affected by arginine starvation (Table 1; Impact > 0.2 and FDR < 0.1). Metabolomic analyses revealed a total number of 32 decreased, 23 increased, and 17 transiently increased or decreased metabolites in 48 h arginine-starved, compared to arginine-replenished, MDA-MB-231 cells (Supplementary Fig. 2A). Among them, an arginine starvation-induced upregulation of 12 intracellular amino acid levels, including the levels of glutamine, asparagine, glycine, and glutamate, was observed, suggesting a substantial re-wiring of amino acid metabolism. Within the TCA cycle, arginine-starved cells transiently decreased then increased the amounts of malate and fumarate, whereas the levels of citrate and α-ketoglutarate were lower at both 24 and 48 h (Fig. 1a, Supplementary Fig. 2A). The consistent decrease in citrate and α-ketoglutarate levels in both tracing and metabolomic analyses further suggests that glutamine metabolism is impaired in arginine-starved cells (Fig. 1c, d). The transient depletion of malate and fumarate, and transient overabundance of aspartate (Supplementary Fig. 2A) suggest that cataplerosis (removal of TCA cycle intermediates) is balanced by anaplerosis (restoration of these intermediates) in arginine-starved cells. In addition, selected glycolytic pathway intermediates, including lactate, fructose 1,6-bisphosphate (Fructose 1,6p), glyceraldehyde 3-phosphate (Glyceraldehyde 3p), and dihydroxyacetone phosphate(DHAP), showed a pronounced decrease in arginine-starved cells (Fig. 1a, Supplementary Fig. 2A).

**Table 1:** Metabolic pathway analysis of MDA-MB231 cells upon arginine starvation

Pathway name	Match status	*p*	FDR	Impact
Alanine, aspartate and glutamate metabolism	6/24	2.35E−05	3.14E−04	0.63153
Arginine and proline metabolism	10/77	2.02E−05	3.14E−04	0.39127
Pyrimidine metabolism	12/60	1.86E−08	6.80E−07	0.38336
Glycine, serine and threonine metabolism	8/48	2.32E−05	3.14E−04	0.37422
Glycerophospholipid metabolism	7/39	4.78E−05	5.46E−04	0.25453
Citrate cycle (TCA cycle)	3/20	0.01418	0.088048	0.20415
Cysteine and methionine metabolism	5/56	0.014308	0.088048	0.1828
Aminoacyl-tRNA biosynthesis	15/75	2.28E−10	1.83E−08	0.16902
Lysine biosynthesis	4/32	0.008846	0.066363	0.15868
Purine metabolism	7/92	0.009125	0.066363	0.14774
D-Glutamine and D-glutamate metabolism	2/11	0.031854	0.1504	0.02674
Nitrogen metabolism	10/39	2.55E−08	6.80E−07	0.0083
Phenylalanine, tyrosine and tryptophan biosynthesis	3/27	0.031961	0.1504	0.008
Galactose metabolism	5/41	0.003792	0.03371	0.00369
Cyanoamino acid metabolism	4/16	6.13E−04	0.006129	0
D-Arginine and D-ornithine metabolism	2/8	0.017056	0.097465	0
Pantothenate and CoA biosynthesis	3/27	0.031961	0.1504	0
beta-Alanine metabolism	3/28	0.035143	0.15619	0
Glycolysis or Gluconeogenesis	3/31	0.045632	0.19213	0

*Note*: The cut-off *p*-value is set at *p* < 0.05. FDR: false discovery rate

We also observed a reduction in the abundance of xanthine, dCTP, thymine, and uracil (Supplementary Fig. 2A) upon arginine starvation. Along this line, we found a reduction in m + 3-labeled serine (Supplementary Fig. 2B) despite quantitative RT-PCR (qRT-PCR) analyses showed an early and transient increase in phosphoglycerate dehydrogenase (*PHGDH*), phosphoserine aminotransferase 1 (*PSAT1*) and phosphoserine phosphatase (*PSPH*) message abundances (Supplementary Fig. 2C) and an increase intracellular steady-state serine amount (Supplementary Fig. 2A). PHGDH, PSAT1, and PSPH are key enzymes in the biosynthesis of serine and glycine, precursor for purines (Supplementary Fig. 2D). It is possible the decreased diversion of glucose to serine is compensated by increased import of serine through transporters or reduced conversion of serine to glycine in one-carbon (1C) metabolism^30^, which is supported by the observation of decreased nucleotides (Supplementary Fig. 2A).

### Mitochondria are the target of arginine starvation

**Fig. 2:**
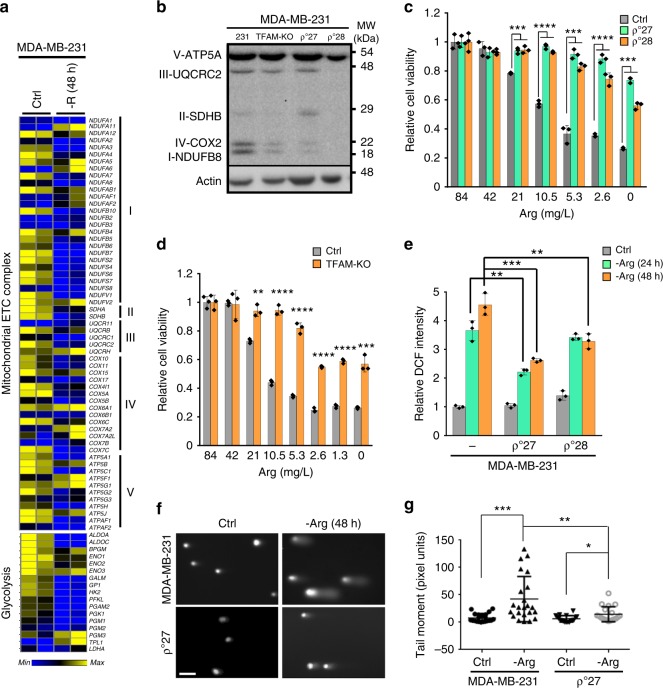
Mitochondria are important targets of arginine starvation. **a** Heatmap of altered expression of mitochondrial complex and glycolysis genes. Gene expression was assessed using RNA-seq in MDA-MB-231 cells maintained in full (Ctrl) and arginine-starved (-R, 48 h) media (*n* = 2 per group). Data are shown in relative reads for each gene. **b** A representative Western blot shows mitochondrial complex protein levels in mitochondria-depleted ρ^0^ and TFAM-KO MDA-MB-231 cells; *n* = 3. The uncropped blot can be found in Supplementary Fig. 10A. **c**, **d** Acid phosphatase (ACP) assays of viability of mitochondria-depleted ρ^0^ (**c**) and TFAM-KO (**d**) cells incubated with decreasing concentrations of arginine for 72 h; *n* = 3. **e** Oxidative stress in mitochondria-depleted ρ^0^ cells after arginine starvation (-Arg). Relative oxidized DCF levels were calculated by designating the value in MDA-MB-231 cells cultured in full medium as 1; *n* = 3. **f**, **g** DNA damage in mitochondria-depleted ρ^0^ cells after arginine starvation. The alkaline comet assay was used for measuring DNA fragmentation. **f** Representative images. Scale bar: 100 µm. **g** The comet tail moment is shown as mean ± S.D.; *n* ≥ 20. For bar graphs, data are shown as mean ± S.D.; *p *<* 0.05; **p *<* 0.01; ***p *<* 0.001; ****p *<* 0.0001

RNA-seq analyses were performed to determine the transcriptome changes in MDA-MB-231 cells in response to 48 h of arginine starvation, compared to the expression levels in cells with arginine-replenished medium. The FPKM were estimated with the selection criteria of *q* value <0.05 and [log2 (fold change)] >1 or <1 for significantly differential expression for up-regulation and down-regulation, respectively. We identified a total of 4330 differentially expressed genes (DEGs) between the arginine-replenished and arginine-starved MDA-MB-231 cells. Among them, 1253 DEGs were up-regulated, while 3077 DEGs were down-regulated. Metascape enrichment analyses (A Gene Annotation and Analysis: http://metascape.org/gp/index.html#/main/step1, updated on 2018-01-01) revealed that portion of down-regulated DEGs were enriched in mitochondrial electron transport complex (ETC) and glycolytic genes (Fig. 2a).

Together with down-regulated mitochondrial metabolites (Fig. 1) and increased ROS production^3,6,25^, we hypothesized that mitochondrial perturbation is a key cellular response to arginine starvation that precedes the initiation of cell death pathways. If so, cells lacking mitochondria should resist the cytotoxic effects of arginine depletion. Therefore, we derived MDA-MB-231 lines that lack functional mitochondria (ρ^0^ cells) by depleting mitochondrial DNA using ethidium bromide^31^ or by CRISPR/Cas10-based knockout (KO) of the mitochondrial transcription factor A (TFAM). Both ρ^0^ cells (2 different clones) and TFAM-KO cells displayed marked decreases in expression of mitochondrial ETC proteins (Fig. 2b). Reduced oxygen consumption rate (OCR), relative to control cells (Supplementary Fig. 3A), and no compensatory glycolysis were observed (Supplementary Fig. 3B). As expected, mitochondria-depleted cells were resistant to the cytotoxic effects of arginine starvation (Fig. 2c, d). To test the possibility that mitochondria-deficient cells should resist arginine starvation-induced ROS generation and DNA damage, we measured the oxidized dichlorofluorescein level in arginine-starved MDA-MB-231 and MDA-MB-231-derived ρ^0^ cells and observed reduced ROS levels in ρ^0^ cells (Fig. 2e). Lastly, we performed alkaline comet assays to examine DNA damage, and observed smaller tail moments, which are indicators of DNA damage, in arginine-starved ρ^0^ cells (Fig. 2f, g). These results support the notion that mitochondria are indispensable for arginine starvation-induced genome instability and cell death.

### Arginine starvation inhibits OxPhos via gene regulation

**Fig. 3:**
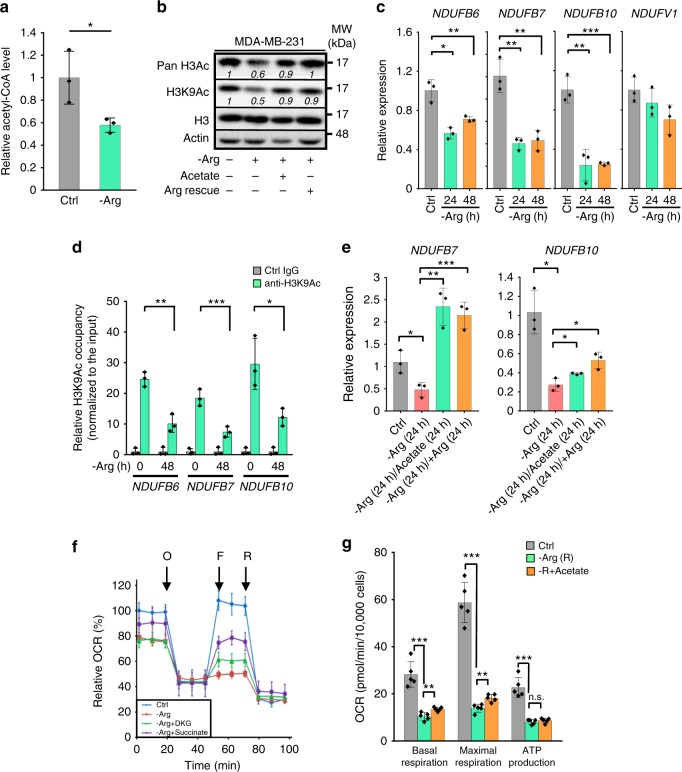
Arginine starvation epigenetically impairs mitochondrial bioenergetics. **a** The relative acetyl-CoA level was assessed in MDA-MB-231 cells after arginine starvation (-Arg) for 48 h by liquid chromatography-mass spectrometry. The relative level was calculated by designating control (Ctrl) cell extracts as 1; *n* = 3. **b** Overall histone H3K9 acetylation (H3K9Ac) after arginine starvation. The cells were cultured in control or arginine-starved medium, as indicated, for 24 h. Acetate (10 mM) or arginine (Arg rescue, 84 mg/L) was added to arginine-starved medium for an additional 24 h. Relative change (over with arginine control) of each sample is calculated by designating the densitometry tracing value in the control as 1 after normalization with actin. The original blot of this panel is included in Supplementary Fig. 10B. **c** qRT-PCR analyses of mitochondrial complex *NDUFB6*, *NDUFB7*, *NDUFB10*, and *NDUFV1* genes upon arginine starvation; *n* = 3. **d** ChIP assay for H3K9Ac occupancy in *NDUFB6*, *NDUFB7*, and *NDUFB10* promoter regions after arginine starvation; *n* = 3. **e** qRT-PCR analyses of mitochondrial Complex I *NDUFB7* and *NDUFB10* expression after arginine starvation and supplementation of arginine (84 mg/L) or acetate (10 mM); *n* = 3. **f** The oxygen consumption rate (OCR) was measured in MDA-MB-231 cells maintained in control or arginine-starved medium in the presence or absence of dimethyl-ketoglutarate (DKG; 10 mM) or succinate (10 mM) using a Seahorse bioanalyzer; *n* = 5. Relative OCR was calculated by designating the basal OCR of cells in full medium as 100%. O: oligomycin; F: FCCP; R: rotenone. **g** Effect of arginine starvation and supplementation with acetate (10 mM) on basal respiration, maximal respiration, and ATP production from Supplementary Fig. 3D; *n* = 5. For bar graphs, data are shown as mean ± S.D.; *p < 0.05; **p *<* 0.01; ***p *<* 0.001

Mitochondria function as signaling organelles through pathways involving ROS production and citrate release, which generates the acetyl-CoA used for protein acetylation^14,15,32^. To address to what extent that arginine depletion disrupts protein acetylation via regulating acetyl-CoA levels, we measured intracellular acetyl-CoA abundance by liquid chromatography-mass spectrometry. As expected, arginine starvation reduced acetyl-CoA production in MDA-MB-231 cells (Fig. 3a). We next investigated the extent to which arginine starvation-mediated down-regulation of acetyl-CoA suppresses histone acetylation, a marker of transcriptional activation. To test this, we measured acetylation of H3K9 (H3K9Ac), a typical transcriptional activation mark, and pan histone H3; and showed that arginine starvation reduces the acetylation of histones (Fig. 3b). Notably, supplementation with acetate, which restores the acetyl-CoA level via acetyl-CoA synthetase short-chain family members 1 and 2 (mitochondrial *ACSS1* and nuclear-cytoplasmic *ACSS2*)^33–35^, or arginine rescued both H3K9 and pan-H3 acetylation (Fig. 3b).

As early as 24 h after arginine starvation, we observed decreased abundance of mitochondrial complex I *NDUFB6*, *NDUFB7*, and *NDUFB10* mRNAs (Fig. 3c). Further, we observed reduced H3K9Ac occupancy in the respective promoter regions of mitochondrial ETC complex I *NDUFB6*, *NDUFB7*, and *NDUFB10* genes under arginine starvation (Fig. 3d). Supplementation with acetate or arginine rescued *NDUFB7* and *NDUFB10* mRNA abundance (Fig. 3e). Together, these data indicate that arginine starvation rapidly and potently induces epigenetic silencing of nuclear-encoded mitochondrial ETC complex genes via histone acetylation.

We next used the Seahorse XF96 to perform metabolic assessments of MDA-MB-231 cells following arginine starvation. Consistent with the decreased ATP concentration we previously observed in arginine-starved MDA-MB-231 cells^6^, arginine starvation blunted the OCR, including ATP-linked respiration (oligomycin sensitive) and reserve capacity (induced by the uncoupler FCCP) (Fig. 3f, g). Arginine starvation also reduced glycolytic flux, as measured by the extracellular acidification rate (ECAR) (Supplementary Fig. 3C), corroborating with the results shown in Fig. 1b and Supplementary Fig. 2A. Given that the α-ketoglutarate level was drastically reduced by arginine starvation (Fig. 1a, c, Supplementary Fig. 2A), we assessed the ability of dimethyl-α-ketoglutarate, a cell-permeable α-ketoglutarate analogue, or succinate, a metabolite downstream of α-ketoglutarate, to rescue arginine starvation-reduced OCR and ECAR. Treatment with dimethyl-α-ketoglutarate or succinate partially restored respiration (Fig. 3f), and rescued glycolysis to an even greater extent (Supplementary Fig. 3C). Previous reports in melanoma and sarcoma cell lines have shown an increase in mitochondrial OxPhos after arginine starvation^24^. This discrepancy is likely due to the length of arginine starvation. In those studies, short-term treatment of cells with ADI-PEG20 decreased mitochondrial OCR. The increased OxPhos was only observed in arginine starvation-resistant cells, in which the cells regained ASS1 expression in a c-Myc-dependent manner^24^. Lastly, we determined the extent to which supplementation with acetate rescues OCR in arginine-starved MDA-MB-231 cells. As shown in Fig. 3g and Supplementary Fig. 3D, acetate, presumably through restoration of acetyl-CoA, partially reversed the effects of arginine starvation on basal and maximal respiration, demonstrating that mitochondrial-nuclear interaction is a key component of arginine starvation-induced metabolic alteration. These evidence together show that arginine starvation results in decreased abundance of key mitochondrial ETC subunits, thereby perpetuating mitochondrial dysfunction.

### Arginine starvation induced ER stress requires mitochondria

**Fig. 4:**
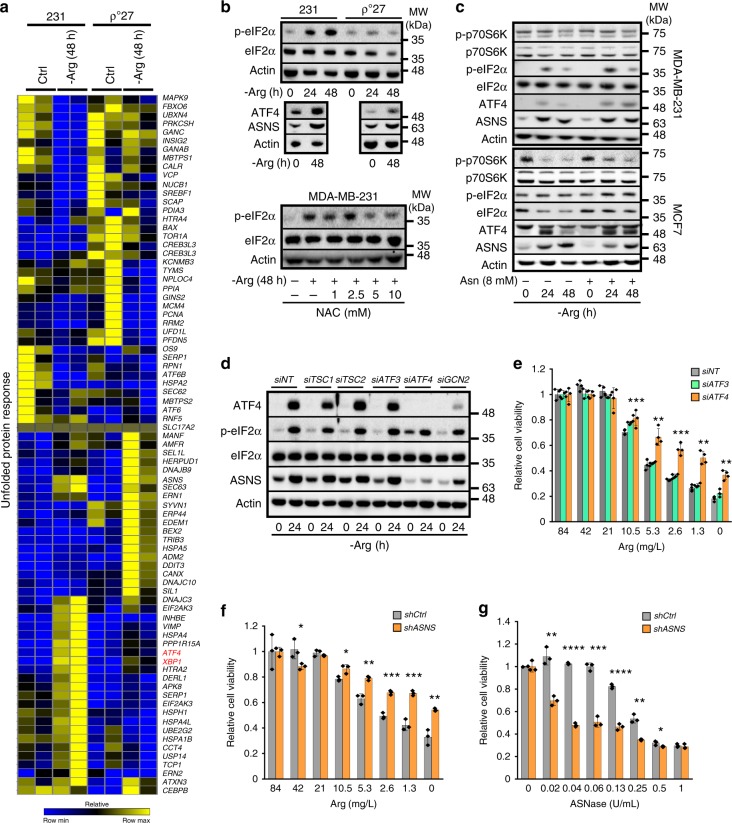
ATF4-dependent ASNS expression links arginine starvation and cell death. **a** Heatmap of the UPR pathway. Gene expression was assessed using RNA-seq in control MDA-MB-231 (231) and mitochondria-depleted MDA-MB-231 ρ^0^ cells treated with full medium (Ctrl) or arginine starvation (-Arg, 48 h). The gene list was established using the UPR RT^2^ profiler PCR array (Qiagen). Data are shown in relative reads for each gene. **b** Representative Western blots of p-eIF2α, ATF4 and ASNS abundance in parental and ρ^0^ cells with or without arginine (-Arg). Treatment with the ROS scavenger, N-Acetyl-cysteine (NAC), dampens phosphorylation of eIF2α. MDA-MB-231 cells under arginine starvation were treated with the indicated concentrations of NAC and subjected to Western blot analyses for p-eIF2α signal. A representative blot is shown here, *n* ≥ 3. The original blots of this panel can be found in Supplementary Fig. 11. **c** Representative Western blots of p-p70S6K, p-eIF2α, ATF4 and ASNS after arginine starvation in both MDA-MB-231 and MCF7 cells; *n* = 3. The unprocessed images are included in Supplementary Fig. 12. **d** Representative Western blots of TSC1, TSC2, ATF3, ATF4, or GCN2 knockdown on ATF4, ASNS, and p-eIF2α induction after arginine starvation (24 h); *n* = 3. The uncropped blots of the panel are included in Supplementary Fig. 13. **e**, **f** MDA-MB-231 cell viability in response to arginine starvation with or without *ATF3/4*-knockdown (**e**; *n* = 4) and *ASNS*-knockdown (**f**; *n* = 3). **g** MDA-MB-231 cell viability under treatment of ASNase, with or without *ASNS* knockdown. For the bar graphs, data are shown as mean ± S.D.; *n* = 3; *p *<* 0.05; **p *<* 0.01; ***p *<* 0.001

After establishing that early changes to mitochondria are important for arginine starvation-induced cytotoxicity, we tested whether arginine starvation induces ER stress, which could exacerbate oxidative stress, and whether mitochondria partake in such an adaptive response^36^. To achieve this goal, we performed RNA-seq analyses on parental and ρ^0^ MDA-MB-231 cells with or without arginine starvation (48 h). Arginine starvation induced the expression of ER stress-responsive genes (the induction of the unfolded protein response [UPR] gene signature), such as *ATF4* and *XBP1*, and the induction of *ATF4* and *XBP1* was mitigated in arginine-starved ρ^0^ MDA-MB-231 cells (Fig. 4a, highlighted in red). We have performed qRT-PCR analyses to validate the induction of *ATF4*, *ASNS* and *XBP1* in arginine-starved MDA-MB-231 cells (Supplementary Fig. 3E).

Activation of the UPR was demonstrated by the induction of prototypical ER stress markers, such as p-eIF2α, ATF4, and ASNS, in response to arginine starvation (Fig. 4b). Consistent with Fig. 4a, arginine-starved ρ^0^ cells displayed reduced p-eIF2α and ATF4 signals upon arginine starvation (Fig. 4b). Also, supplementation with the antioxidant N-acetylcysteine (NAC) partially reduced the p-eIF2α signal in parental cells (Fig. 4b), supporting a role for mitochondrial ROS (Fig. 2e) in the link between arginine starvation and ER stress. To characterize the underlying mechanism, we examined whether cells resistant to other ER stressors, such as tunicamycin (TM), are resistant to arginine starvation-induced cell death. We established TM-resistant MDA-MB-231 cells (Supplementary Fig. 3F) and found that these cells were also resistant to the ER stress inducer thapsigargin (TG) (Supplementary Fig. 3G), but not to arginine starvation (Supplementary Fig. 3H). This result suggests that classical ER stress is not the major factor contributing to arginine starvation-induced cytotoxicity.

Next, we used MCF7 cells, resistant to arginine starvation-induced cytotoxicity^6^, to investigate the effect of arginine starvation-induced ER stress in ASS1-high context. Arginine is one of three amino acids that can directly activate mTORC1^37–41^. We confirmed that arginine starvation (24 h) diminished mTOR signaling (evidenced by decreased mTOR downstream effector p70S6K phosphorylation) and increased ATF4 and ASNS in both ASS1-low MDA-MB-231 and ASS1-high MCF7 cells (Fig. 4c), suggesting that the induction of ATF4-ASNS represents a common response to extracellular arginine starvation. Lastly, treatment of MHY1485^42^, a mTOR activator, significantly improved the viability of arginine-starved MDA-MB-231 (Supplementary Fig. 4A). In parallel, rapamycin, a mTOR inhibitor, notably reversed the rescuing effect of arginine supplementation on cell proliferation fitness in arginine-starved (48 h) MDA-MB-231cells (Supplementary Fig. 4B) and overcame the inhibitory effect by arginine on expression of *ASNS* and *ATF4* (Supplementary Fig. 4C), markers for ER stress. Together, our data support that mTOR is not only acts as a sensor of cell arginine state^43^, but also is intimately involved in regulating the cell fitness in response to arginine starvation.

Asparagine supplementation partially restored the suppression of p70S6K phosphorylation, a hallmark of mTOR activation, and delayed ASNS induction in response to arginine starvation (24 h) in MCF7, but not MDA-MB-231, cells (Fig. 4c), suggesting that there are different thresholds for exogenous arginine, dependent on the ASS1 level, to activate mTOR in these two distinct ASS1-contexts. Moreover, asparagine supplementation did not reduce the arginine starvation-induced p-eIF2α signal in MDA-MB-231 cells (Fig. 4c), and arginine starvation did not induce p-eIF2α in MCF7 cells (Fig. 4c).

To identify the key signaling molecule(s) responsible for arginine starvation-induced UPR, we used siRNA gene knockdown to probe the involvement of TSC1, TSC2, ATF3, ATF4, and GCN2 in the stress response to arginine starvation. We first confirmed that arginine starvation increased the abundance of ATF4, p-eIF2α, and ASNS in siNT (control siRNA)-transfected MDA-MB-231 cells (Fig. 4d, left two lanes). Individual knockdown of ATF4 or GCN2, but not TSC1/2 or ATF3, attenuated the induction of ATF4 and ASNS in arginine-restricted cells (Fig. 4d). This was further exemplified by our observation that knockdown of ATF4, but not ATF3, rendered the cells resistant to arginine starvation (Fig. 4e). Unexpectedly, knockdown of ASNS rescued the viability of arginine-starved MDA-MB-231 cells (Fig. 4f), but sensitized cells treated with asparaginase (Fig. 4g, a control for ASNS function). ATF3-knockdown cells exhibited similar arginine sensitivity to siNT-cells (Fig. 4e). We then further confirmed that the unfolded protein response (UPR) activated by arginine starvation then excised 26 nucleotides from the mRNA of unspliced X-box binding protein 1 (*XBP1u*), resulting in a frame shift to produce the mature, spliced XBP1 (*XBP1s*)^44^, in both arginine-starved MDA-MB-231 and BT-549 cells, as in response to ER stress induced by either Tm or Tg (Supplementary Fig. 4D). Lastly, knockdown of *XBP1* by two different targeting siRNAs, compared to scrambled siRNA (siCtrl), increased cell viability, as siATF4 and siASNS did, in arginine-starved MDA-MB-231 cells (Supplementary Fig. 4E). It is likely that arginine starvation induces a non-canonical ER stress response, leading to cytotoxicity in ASS1-low cells.

### Aspartate rescues metabolism after arginine starvation

**Fig. 5:**
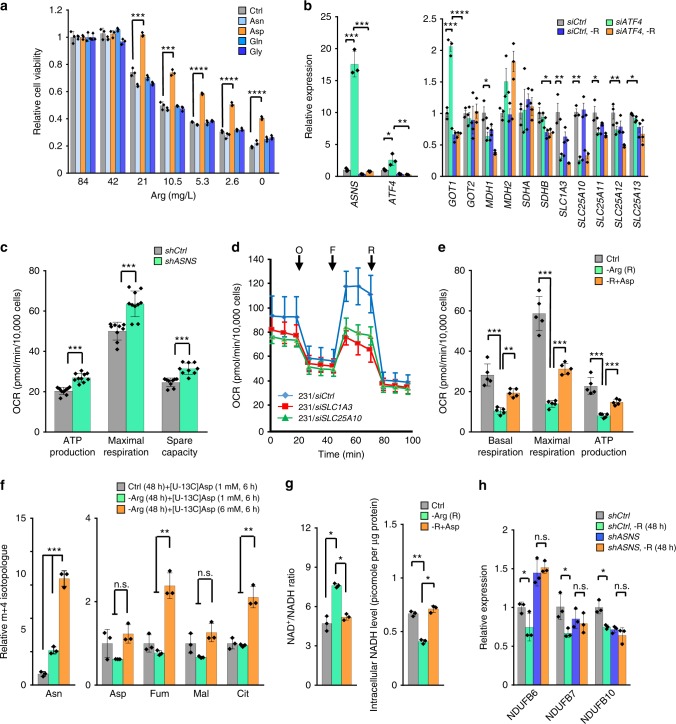
Aspartate rescues the viability of arginine-starved cells. **a** Cell viability of MDA-MB-231 cells after arginine starvation with or without supplementation of asparagine, aspartate glutamine or glycine (10 mM each) for 72 h; *n* = 3. **b** qRT-PCR analyses of *ASNS*, *ATF4* and the mRNA abundance of the key components of the malate-aspartate shuttle after arginine starvation (-R; 24 h) in siCtrl- and siATF4-cells; *n* = 3. **c** Effect of ASNS-knockdown on basal respiration, maximal respiration, and ATP production measured in Supplementary Fig. 5C; *n* = 9. **d** OCR was measured in SLC1A3- or SLC25A10-knockdown cells; *n* = 4. O: oligomycin; F: FCCP; R: rotenone. **e** Effect of arginine starvation and replenishment with aspartate (10 mM) on the basal respiration, maximal respiration, and ATP production measured in Supplementary Fig. 5D; *n* = 5. **f** MDA-MB-231 cells were cultured with [U-^13^C]aspartate for 6 h after 48 h of incubation in arginine-depleted (-Arg) or full (Ctrl) media. The relative aspartate-derived m + 4 fractions of intracellular asparagine (Asn), aspartate (Asp), fumarate (Fum), malate (Mal), and citrate (Cit) were measured with gas chromatography mass spectrometry. The relative m + 4 isotopologue was calculated by designating the respective mean value in MDA-MB-231 ctrl cells as 1; *n* = 3. **g** NAD^+^/NADH ratio and NADH abundance in arginine-starved MDA-MB-231 cells with or without aspartate (10 mM) supplementation; *n* = 3. **h** qRT-PCR analyses of *NDUFB6*, *NDUFB7*, and *NDUFB10* expression in arginine-starved *shCtrl* and *shASNS* cells (48 h). The values are normalized to the 18 S rRNA levels and the mean expression level in the control cells; *n* = 3. For bar graphs, data are shown as mean ± S.D.; n.s., not significant; *p *<* 0.05; **p *<* 0.01; ***p *<* 0.001; ****p *<* 0.0001

It was previously demonstrated that asparagine rescues the survival of glutamine-deprived cells^45^ by functioning as a counter ion in the import of extracellular amino acids to sustain mTOR activation and protein translation^46,47^. However, it remains to be determined whether asparagine rescues the viability of arginine-starved cells in an analogous manner, or whether ASNS plays other regulatory role(s) in response to arginine starvation. To test these two possibilities, we assessed the extent to which supplementation with the amino acids aspartate, asparagine, glutamine, or glycine, rescues cell viability after arginine starvation. Among these amino acids, only supplementation with aspartate preserved cell viability during arginine starvation (Fig. 5a). Asparagine supplementation did not rescue cell viability (Fig. 5a), which is consistent with our finding that impairing asparagine biosynthesis benefited cell viability after arginine starvation (Fig. 4g). Together, we conclude that ASNS has no rescuing effect on arginine-dependent cell viability.

Aspartate, a non-essential amino acid, is synthesized de novo by transamination of oxaloacetate in either the mitochondria or the cytoplasm by GOT1/2, which encodes aspartate aminotransferase, or is transported into the cells by SLC1A3, the glutamate-aspartate transporter^48^. GOT1/2 are important components of the malate-aspartate shuttle, which reversibly transfers electrons from the cytoplasm into mitochondria matrix (Supplementary Fig. 5A). Therefore, we focused on the malate-aspartate shuttle (Supplementary Fig. 5A), as aspartate supports mitochondrial OxPhos^49^. We used qRT-PCR to measure the mRNA abundance of key components in the malate-aspartate shuttle. Consistent with the induction of *ASNS*, *GOT1* was induced by arginine starvation in an ATF4-depedent manner (Fig. 5b). In contrast, we observed the down-regulation of *MDH1* (malate dehydrogenase 1), *SLC1A3*, *SLC25A10* (mitochondrial dicarboxylate transporter for malate and succinate) and *SLC25A11* (mitochondrial oxoglutarate/malate carrier) in an ATF4-independent manner in arginine-starved MDA-MB-231 cells. Moreover, knockdown of *ASNS* suppressed the effect of arginine starvation on expression of *GOT1*, *MDH2*, and *SLC25A11*, compared to shCtrl*-*cells (Supplementary Fig. 5B).

Next, we hypothesized that arginine starvation-induced ASNS and GOT1 consume aspartate, thereby reducing the availability of aspartate, which results in mitochondrial dysfunction. To understand if the aspartate levels regulate mitochondrial function, we knocked-down ASNS, SLC1A3, or SLC25A10 and measured OCR in these cells. These genes were chosen because that their message abundances were regulated by arginine starvation and that their encoded proteins are involved in maintaining aspartate homeostasis. As shown in Fig. 5c and Supplementary Fig. 5C, ASNS knockdown alone increased the basal and ATP-linked respiration and reserve capacity in MDA-MB-231 cells. Consistent with this, knockdown of SLC1A3 or SLC25A10 alone had an opposite effect on OCR (Fig. 5d). These observations indicate that an inefficient malate-aspartate shuttle suppresses mitochondrial function. Indeed, aspartate supplementation increased the basal and maximal respiration and ATP production in arginine-starved MDA-MB-231 cells (Fig. 5e, Supplementary Fig. 5D). In addition, pyruvate carboxylase, which catalyzes the production of oxaloacetate as part of anaplerosis for the TCA cycle^50,51^, could contribute to aspartate homeostasis. However, co-treatment with pyruvate, unlike with DKG, failed to overrule the anti-proliferative effect of arginine starvation (Supplementary Fig. 5E).

To further understand the role of aspartate during arginine starvation, we analyzed the mass isotopologue distribution of the principal aspartate catabolism intermediates in arginine-starved (48 h) MDA-MB-231 cells using [U-^13^C] aspartate as a tracer during the last 6 h of starvation (Supplementary Fig. 6A). Figure 5f reveals that aspartate, malate, and fumarate enrichment were lower in arginine-starved cells, and there was a robust increase in asparagine enrichment compared to cells maintained in arginine-containing medium, indicating that arginine starvation-induced ASNS converts aspartate to asparagine. Moreover, supplementation with a bolus of aspartate (6 mM) doubled the m + 4 fraction of fumarate and citrate (Fig. 5f, Supplementary Fig. 6B), supporting the possibility that aspartate rescues mitochondrial function in arginine-starved cells though the malate-aspartate shuttle.

Mitochondrial NADH pools tend to be oxidized by ETC Complex I and NAD^+^/NADH balance is one of the key regulators of energy metabolism, including mitochondrial fitness. We have shown that arginine starvation impairs NADH reducing equivalents transporters in-and-out of mitochondria (Fig. 5b). To determine the extent to which arginine starvation suppresses OxPhos via NADH reduction, we measured the intracellular levels of NAD^+^ and NADH after arginine starvation. Arginine starvation disrupted the NAD^+^/NADH ratio and, interestingly, the effects of arginine starvation on total NADH levels were reversed by aspartate (Fig. 5g). This indicates that not only is NADH transport into mitochondria generally defective in arginine-starved cells, but also the overall NADH production is compromised due to lack of aspartate. This observation was further validated by an independent measurement of the glutamate-stimulated NADH autofluorescence intensity. As expected, arginine starvation reduced glutamate-stimulated NADH autofluorescence, while aspartate supplementation or *ASNS* knockdown reversed the reduction (Supplementary Fig. 6C). To further validate that arginine starvation reduces NADH abundance, we assessed the effect of metformin, a known Complex I inhibitor^21^, on sensitizing cells to arginine starvation as a separate paradigm. Metformin sensitized MDA-MB-231 cells to reduced glucose (Supplementary Fig. 6D), serving as a control. Consistent with the notion that metformin impairs mitochondrial function, knockdown of ASNS abolished the sensitizing effect of metformin (Supplementary Fig. 6D). As shown in Supplementary Fig. 6E, metformin sensitized, albeit to a lesser extent, cells to reduced arginine level. In addition, ASNS-knockdown, at least in part, prevented the down-regulation of mRNA abundance of several Complex I genes (*NDUFB6*, *NDUFB7*, and *NDUFB10*) in arginine-starved MDA-MB-231 cells (Fig. 5h), supporting that ER stress-induced ASNS suppresses the expression of nuclear genes encoding mitochondrial ETC genes.

### Cytotoxicity of arginine starvation in ASS1-low cancer cells

To directly test the biological role of aspartate in the context of arginine starvation, we measured nucleotide pools in arginine-starved (48 h) or untreated-control MDA-MB-231 cells, with or without aspartate supplementation. In agreement with the concept that aspartate is spared from nucleotide biosynthesis in arginine-starved cells, arginine starvation decreased the intracellular dATP and dTTP pools, and aspartate supplementation restored the nucleotide pools (Supplementary Fig. 7A). Next, we found that adding dNTP rescued the proliferation defect of arginine starvation (Supplementary Fig. 7B). We measured the effect of orotate, an aspartate downstream pyrimidine precursor (Supplementary Fig. 7C) on the viability of arginine-starved cells. As expected, supplementation with orotate rescued the viability of arginine-starved MDA-MB-231 cells (Supplementary Fig. 7D). The reduced dNTP pools may affect DNA repair, which could explain the resultant DNA damage and genome instability we observed following arginine starvation (Fig. 2f).

Next, ASS1-deficient BT-549 and MDA-MB-435 breast cancer cells were used to investigate whether the metabolic alterations identified were a MDA-MB-231-specific phenomenon or a general characteristic of arginine auxotrophy. We first confirmed that arginine starvation induced ROS production (Supplementary Fig. 8A) and inhibited cell proliferation (Supplementary Fig. 8B) in ASS1-low BT-549 cells, similar to MDA-MB-231 cells. In addition, knockdown of ASNS rescued the viability of arginine-starved BT-549 and MDA-MB-435, as expected based on our results using MDA-MB-231 cells (Supplementary Fig. 8C). The combination of ASNase, converting asparagine to aspartate, and exogenous asparagine rescued the viability of arginine-starved BT-549 cells (Supplementary Fig. 8D). Likewise, aspartate was more effective than acetate in mitigating the damage by arginine starvation on mitochondrial function in BT-549 cells (Supplementary Fig. 8E) and rescued the total NAD^+^/NADH ratio (Supplementary Fig. 8F). We also confirmed that NADPH abundances were decreased in both arginine-starved MDA-MB-231 and BT-549 cells (Supplementary Fig. 8G) and MHY1485, a mTOR activator, improved the viability of arginine-starved BT-549 cells (Supplementary Fig. 8H). Lastly, supplementation with orotate rescued the viability of arginine-starved BT-549 and MDA-MB-435 breast cancer cells (Supplementary Fig. 8I, J).

### Arginine starvation restricts in vivo breast tumor growth

**Fig. 6:**
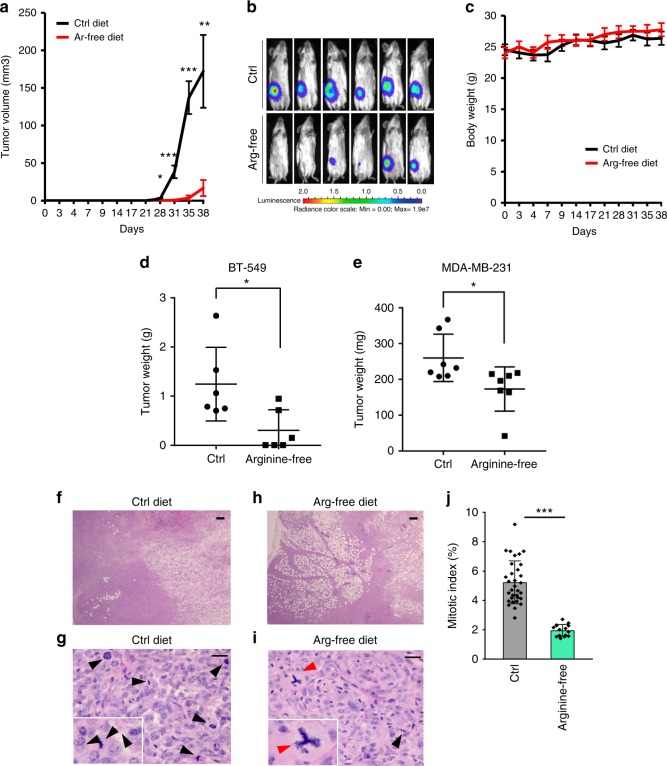
Arginine starvation reduces tumor size in a xenograft model. **a**, **b** The effect of an arginine-free diet, compared to the matched control diet (Ctrl), on orthotopically xenografted luciferase-tagged breast cancer BT-549 cells was measured by tumor volume (**a**) and bioluminescence imaging (**b**). **c** Effect of an arginine free-diet on mouse body weight. **d**, **e** The weights of tumors dissected from BT549 cell xenografted mice (**d**) and MDA-MB-231 cell xenografted mice (**e**) fed with either control or arginine-free diet (Mice failed to develop tumor after arginine starvation was assigned value “0” to for analysis; *n* = 6). **f**–**i** Representative histological analysis (hematoxylin and eosin) of tumors harvested from mice maintained on a control diet (**f**, **g**) and arginine-free diet (**h**, **i**) at day 39 post-tumor cell orthotopic implantation. Arrowheads indicate mitotic cells. Expanded view of the mitotic cells (black arrowheads) and multi-polar anaphase cell (red arrowhead) are shown; scale bar: 50 µm (**f**, **h**); scale bar: 500 µm (**g**, **i**). **j** Mitotic cells were quantified from five high-power fields (HPF) from each group. Data are shown as mean ± S.D.; *p *<* 0.05; **p *<* 0.01; ***p *<* 0.001; ****p *<* 0.0001

To test the effect of arginine starvation on tumor cells in vivo, we examined tumor growth in ASS1-low BT-549 and MDA-MB-231 breast cancer cell xenograft model subjected to dietary arginine restriction. Arginine-free diet significantly retarded tumor growth of orthotopically xenografted BT-549 breast cancer cells in vivo, as measured by volume (Fig. 6a) and luciferase imaging (Fig. 6b). The arginine-free diet had no obvious adverse effect on body weight (Fig. 6c). While we observed a marked decline of tumor weights in BT-549 tumor xenografts (Fig. 6d), a less pronounced, albeit significant, tumor weight reduction was observed in MDA-MB-231 xenografts (Fig. 6e). The activated KRAS mutation in MDA-MB-231 cells^52^ could, at least in part, account or the lack of robust response of MDA-MB-231 cells to arginine starvation in vivo (Fig. 6e), as previously implicated in the resistance to serine/glycine starvation in vivo^53^. In addition, the higher basal ROS levels in BT-549 cells (Supplementary Fig. 8A) could render them more vulnerable to arginine starvation. To determine the effects of arginine-free diet on tumor volume, the BT-549 xenografted tumors were serially sectioned and analyzed histologically. Consistent with the cell culture model (Supplementary Fig. 8B), lack of arginine decreased BT-549 cell survival and proliferation, as indicated by reduced tumor cell infiltration (Fig. 6f, h) and the presence of fewer mitotic cells (Fig. 6g, i, j). The tumors from mice fed an arginine-free diet (Fig. 6h), showed a large area of residual fat (left field, ~50%); however, the control tumor, exhibited much less residual fat (Fig. 6f, left field, ~5%). We also noticed the presence of multipolar anaphases, known to result in eventual cell death^54^ in tumor sections from mice fed arginine-free diets (Fig. 6i, red arrow). Overall, these results indicate that dietary arginine restriction can suppress the growth of ASS1-low tumors in vivo.

## Discussion

Three main findings reported herein led us to conclude that mitochondria are the critical targets linking arginine starvation to cell death: (1) arginine starvation modulates mitochondrial ETC gene expression; (2) arginine starvation induces ATF4-ASNS, which diverts cellular aspartate toward increased asparagine and suppresses the aspartate-malate shuttle; and (3) mitochondria-deficient cells are resistant to the effects of arginine starvation (Supplementary Fig. 9). This metabolic shift reduces mitochondrial OxPhos and impedes nucleotide biosynthesis. Further, our evidence shows that unresolved ATF4-ASNS-dependent ER stress reduces the viability of arginine-starved cells. Our report emphasizes that arginine starvation induces a complicated intracellular response, with both dysregulated ROS and acetyl-CoA production transmitting information about arginine sufficiency (or lack thereof) to the genome. We established that arginine is essential for proper mitochondrial function, which is critical for cellular acetyl-CoA level to maintain the H3K9Ac occupancy of key mitochondrial ETC complex genes. We further demonstrate that aspartate insufficiency, resulting from arginine starvation-induced ASNS overexpression, is an important vulnerability in arginine-starved ASS1-low cells. Because ASNS overexpression induces a metabolic shift, aspartate is conceivably essential for proper mitochondrial respiration and genome integrity in arginine-starved cells.

It is intriguing that the intracellular aspartate levels, albeit inversely correlated with malate levels, transiently increased during arginine starvation (Fig. 1a). Brisoy et al. reported that GOT1 generates aspartate from glutamine decarboxylation, instead of consuming it, during ETC inhibition^55^. This could explain why aspartate was not markedly reduced after arginine starvation, as *GOT1* is markedly induced in arginine-starved cells. In addition, the authors showed that overexpression of SLC1A3 alone rescued the proliferation inhibition caused by ETC inhibition. Our data showed that *SLC1A3* expression was significantly attenuated in arginine-starved cells. However, despite *GOT1* induction, the inability of pyruvate to rescue arginine-starved cells suggests that pyruvate carboxylation to oxaloacetate, which is used by GOT1 to generate aspartate, is likely suppressed in arginine-starved MDA-MB-231 cells. We propose that MDA-MB-231 cells similar to many other cell types rely more on the malate-aspartate shuttle than pyruvate carboxylation to replenish oxaloacetate^56^. Therefore, the down-regulation of *MDH1* and *SLC25A10* by arginine starvation (Fig. 5b) could override the net influx of NADH into mitochondria. These data together suggest that arginine starvation, at least in part, reduces OxPhos, NADH, and aspartate availability through ASNS induction (Supplementary Fig. 9). In the current study, we demonstrated that arginine-starved cells fail to reserve aspartate, resulting in metabolic inflexibility. To support the concept that the aberrant secondary cellular responses to disrupted metabolic homeostasis may contribute to cell death, we showed that supplementation of aspartate or orotate, which alleviate the deficiencies caused by secondary cellular responses to arginine starvation, also alleviate the arginine starvation phenotypes^3,6,25^.

Two recent studies^55,57^ raise the possibility that the impairing effects of arginine starvation on NADH production may be critical for cytotoxicity. They demonstrated that the uncoupling agent FCCP, which dissipates the electrochemical gradient across the mitochondrial membrane, rescues the inhibition of cell viability by oligomycin, an ATP synthase inhibitor, by resetting the NAD^+^/NADH ratio. The authors suggested that uninterrupted NADH flux, allowing for transfer of electrons through the ETC, is indispensable for cell survival and proliferation. Therefore, it is possible that arginine, at least in part, stimulates mitochondrial respiration, which is required for aspartate anabolism and catabolism in proliferating cells. Along these lines, we showed that sustained arginine starvation impairs the mitochondrial ETC and disables the aspartate-mediated compensatory augmentation of mitochondrial function.

In conclusion, these metabolic disruptions clarify how cancer cells respond to arginine starvation through an ATF4-ASNS axis to switch from an adaptive UPR from ER stress to a non-canonical ER stress response and cell death. As a result, cells subjected to prolonged arginine shortages cannot stably supply NADH to fuel mitochondrial ETC Complex I while preventing the lethal cellular stresses associated with starvation. We further demonstrated that dietary limitation of arginine can be effective in retarding the growth of arginine auxotrophic (ASS1-low) breast cancer BT-549 cells. Knockdown of ASNS, an enzyme responsible for *de novo* asparagine synthesis from aspartate and glutamine, allows arginine auxotrophic cancer cells to resist arginine depletion by restricting aspartate consumption. The present work provides insights into a mechanistic link between cell signaling pathways and metabolic pathways in arginine-starved, ASS1-low breast cancer cells. In tumors poorly supplied with nutrients, slowing anabolism can paradoxically promote growth^58,59^; therefore, it is possible that promoting anabolism will maximize the therapeutic potential of arginine starvation in arginine auxotrophic cancer. Our results clarify the metabolic phenotype of arginine starvation and emphasize the importance of activating multiple stress pathways to confer the maximum anti-cancer benefit of arginine starvation.

## Methods

### Cell lines and reagents

All cells were passaged for fewer than 20 passages from liquid N_2_. MDA-MB-231, MCF7, HEK293T, MDA-MB-435, and BT-549 were originally obtained from the American Type Culture Collection (ATCC). MDA-MB-231, MCF7, BT-549 and HEK293T cells were maintained in DMEM (Corning, 10-013-CV); MDA-MB-435 cells in DMEM/F12 (Corning, 10-090-CV) supplemented with 10% fetal bovine serum (Gibco, 10437028) and 1% antibiotics-antimycotics (Gibco, 15240062). The mitochondria-depleted MDA-MB-231 ρ^0^ cell line was a gift from Dr. Kyle Miller (The University of Texas at Austin) and has been maintained in DMEM supplemented with 10% FBS, sodium pyruvate (1 mM), uridine (50 µg/mL), and ethidium bromide (50 ng/mL). sh*ASNS* and sh*Control* cells were maintained in the presence of puromycin (2 µg/mL). The tunicamycin-resistant MDA-MB-231 cells were selected with escalating concentrations of tunicamycin from 20 nM to 2 µM, modified from the previously described method^60^. The cells were initially cultured in DMEM containing tunicamycin at the IC_50_ (20 nM), and then were repeatedly subcultured at a ratio of 1:15 with doubled previous concentrations of tunicamycin when the cell confluency reached 90%. Finally, a pool of resistant MDA-MB-231 cells was selected and maintained in DMEM with tunicamycin (2 µM). Arginine-starved DMEM (Gibco, A14431-01) was supplemented with 10% dialyzed fetal bovine serum (Gibco, 26400), L-glutamine (4 mM, Gibco, 25030081), and L-lysine (0.8 mM, Sigma-Aldrich, L5501). Dimethyl-2-ketoglutarate (DKG) (349631), tunicamycin (T7765), thapsigargin (T9033), puromycin (P8833), N-acetyl-L-cysteine (A7250) asparagine (A4159), and aspartic acid (A8959) were obtained from Sigma-Aldrich.

### Metabolomics

The experiments were performed as described61. Briefly, the experimental cells were grown to 70% confluence and three biological replicates, consisting of equal numbers (approximately 2 × 106) of cells, were included in each group. Metabolite extraction, mass spectrometry analysis, and data analysis were carried out at the UCLA Metabolomics center. To extract the intracellular metabolites, the cells were briefly rinsed with cold ammonium acetate (150 mM, pH 7.3), and scraped into cold 80% methanol (1 ml, −80 °C on dry ice). The cell suspensions were thoroughly mixed in microcentrifuge tubes with addition of norvaline (10 nM, as an internal standard) followed by centrifugation (18,000×*g*) at 4 °C. The supernatant was dried under vacuum in a new glass tube and resuspended in 50% acetonitrile (ACN, 50 µl). Five microliter of the resolved samples were applied for analysis. Chromatographic separation was performed on an UltiMate 3000RSLC system (Thermo Scientific) equipped with a Luna NH2, 150 mm × 2 mm column (from Phenmenex), using the following gradient: (A) NH_4_AcO (5 mM, pH 9.9) and (B) ACN; in 18 min, the gradient starts from 15% (A) to 90% (A), followed by an isocratic step for 9 min and reverse to the initial 15% (A) for 7 min. Eluting compounds were detected by a Q Exactive mass spectrometer (Thermo Scientific) with polarity switching (+3.50 kV/−3.50 kV) in full scan mode with an m/z range of 65–975. Detected Metabolites were identified by mass measurements (≤ 3ppm) and retention times using TraceFinder 3.3. The statistical analyses were performed using 2-way ANOVA. The metabolites that were significantly altered (*p* < 0.05) in the metabolomics study were submitted for metabolic pathway and impact analysis. The analysis was performed at the MetaboAnalyst 3.0 web server (http://www.metaboanalyst.ca^62^) using a hypergeometric test for overrepresentation analysis and relative-betweenness centrality based on the KEGG database (Release 82.1). For acetyl-CoA quantification, an equal number of cells from each group was snap-frozen in liquid nitrogen after harvesting. The frozen cell pellet was thawed on ice, subjected to metabolite extraction twice with 100% methanol (HPLC grade) and once with distilled water, and dried at 30 °C. Metabolite identification was conducted using a MALDI-TOF/TOF 5800 system (AB SCIEX) mass spectrometer. The mass-detecting range was from 50 to 1,000 Da, with a focus at 500 Da. Metabolite peaks were identified based on the mass-to-charge (m/z) ratio referenced from multiple Massbank databases (MoNA http://mona.fiehnlab.ucdavis.edu/, MassBank http://www.massbank.jp/?lang=en, and NORMAN MassBank http://massbank.eu/MassBank/) and quantified by peak intensities. The average peak intensities for each metabolite were calculated from seven mass spectra generated from the same sample and presented as relative fold change to the control group. The peak intensity of the CHCA (*m*/*z* = 188) matrix was used for signal normalization control for sample loading and excitation efficiency.

### Metabolic stable isotope tracing

[U-^13^C]aspartate tracer (CLM-1801-H-0.25) were purchased from Cambridge Isotope Labs Inc. Stable isotope tracing experiments to determine isotopologue distributions in soluble metabolites were performed as described previously^63,64^. The control and arginine-starved (48 h) MDA-MB-231 cells were incubated in [U-^13^C]aspartate containing medium during the last 6 h of arginine starvation. The intracellular metabolites were harvested, derivatized and measured with gas chromatography mass spectrometry. For simplicity, only the m + 4 isotopologue of metabolites were shown as the products of the first-round aspartate catabolism.

For ^13^C-glucose labeling experiments, cells were cultured in full growth medium or arginine depleted medium for 48 h. [U-^13^C]glucose (10 mM) tracer medium with or without arginine were applied to cells for 6 h prior to harvesting. Isotope-labeled aspartate and glucose tracer medium was prepared from phenol red-free, glucose-free, arginine-free, lysine-free, glutamine-free DMEM (Gibco) supplemented with 10% dialyzed FBS, DMEM-levels of L-lysine and L-glutamine.

### dNTP measurement

Cells (1 × 10^6^) were extracted with freon/trioctylamine (55%:45%) and vortexed, followed by centrifugation at 20,000×*g* for 5 min. The supernatant was transferred into a new tube. Determination of the dNTP pool in each supernatant was based on DNA polymerase-catalyzed incorporation of radioactive dNTP into the synthetic oligonucleotide template as described^65^. The reaction mixture (100 μL) contained 40 mM Tris-HCl (pH 7.4), MgCl_2_ (10 mM), dithiothereitol (5 mM), oligonucleotide template (0.25 μM), RNase A (1.5 μg), ^3^H-dATP (for dTTP, dCTP, and dGTP) or ^3^H-dTTP (for dATP) (0.25 μM), Klenow fragment (0.2 units, NEB, for dTTP), Klenow fragment (0.025 units, NEB, for dATP), or Taq polymerase (2 units, Zgene, for dCTP and dGTP) and 16 μL cell extract. Incubation was carried out for 60 min at 37 °C for dTTP and dATP, or at 48 °C for dCTP and dGTP, and reaction mixtures were spotted onto Zeta-probe blotting membrane (Bio-Rad). The membranes were dried, washed with 5% Na_2_HPO_4_ (3 × 5 min), and rinsed once with distilled water and once with 95% ethanol. After drying, 3 mL of Scintillation Cocktail (Perkin Elmer Ultima Gold) was added to the membranes. Radioactivity was measured in a liquid scintillation counter (HIDEX 300 SL).

### Comet assay

The alkaline comet assays were done using the CometAssay kit (4050-050-K) with the CometAssay Eletrophoresis System II (4250-050-ES) from Trevigen following the manufacturer’s instructions. Briefly, the cells were diluted to a concentration of 10^5^ cells/ml, and 25 µl of the cells were mixed with 250 µl of the LMAgrose (Trevigen, 4250-050-02 LMAgrose,). 50 µl of the mixture was applied to the provided slides. The gel spots were allowed to solidify at 4 °C in the dark and subsequently incubated with lysis solution (Trevigen, 4250-050-01) with 1% DMSO at 4 °C overnight. The slides were then immersed in alkaline unwinding solution (200 mM NaOH, 1 mM EDTA, pH > 13) for 1 h at 4 °C and subjected to electrophoresis in alkaline buffer (200 mM NaOH, 1 mM EDTA, pH > 13). The slides were washed twice with dH_2_O and once with 70% ethanol after electrophoresis and air dried at 37 °C. DAPI mounting solution (Life Technologies, P36935) was used to visualize the DNA fragments. Analysis and quantification of DNA damage (Tail moment = Tail length × Tail% of DNA) were performed using fluorescence microscopy and the OpenComet module of Image J (NIH).

### CRISPR knockout of TFAM

Guide RNAs were designed using the online CRISPR design tool (http://crispr.mit.edu/). The forward primer (5′-CACCGTGGCGTTTCTCCGAAGCATG-3′) and reverse primer (5′-AAACCATGCTTCGGAGAAACGCCAC-3′) were cloned into lentiCRISPR v2 (Addgene, 52961) containing the entire guide RNA scaffold, as described previously^66^. Lentiviral medium was produced and applied to MDA-MB-231 cells. Clonal cell lines were isolated by serial dilution and confirmed by PCR sequencing and Western blot. Off-target activity was analyzed by deep sequencing and aligned using BLAST (NIH). TFAM-KO cells were cultured in DMEM supplemented with 10% FBS and uridine (5 µg/ml).

### RNA extraction, RNA sequencing, and qRT-PCR

Total RNA was extracted from cells using the TRIzol reagent (Thermo Fisher, 16696026) for RNA-seq or RNeasy mini kit (Qiagen, 74104) for qRT-PCR. RNA-seq was conducted by the Integrative Genomics Core at City of Hope and the data are available in the Gene Expression Omnibus (GEO) database (GSE104105). qRT-PCR was performed as described previously^3,6^. The primers used in this study are listed in Supplementary Table 1. qRT-PCR data were analyzed using 2^ΔΔCt^ method and the relative mRNA abundance was calculated, after normalization to 18S RNA, by designating the mean mRNA abundance in the control condition as 1. At least 3 biological replicates were included for each analysis.

### Chromatin immunoprecipitation and PCR (ChIP-PCR)

The ChIP assay was performed according to the method described in ref.^67^ with some modifications. Briefly, the cells were cross-linked with 1% formaldehyde (Sigma-Aldrich, 47608) for 10 min at room temperature and quenched with 125 mM glycine for another 10 min. The cells were washed once with ice-cold PBS, scraped off the plate, and sonicated using a Bioruptor Pico (Diagenode) with 5 cycles of 30 s on and 30 s off at a high power setting. Sonicated samples were cleared by centrifugation at 14,000×*g* at 4 °C for 10 min. The supernatant of each sample was first cleared by incubating with pre-blocked Protein G PLUS-agarose beads (Santa Cruz, sc-2002) at room temperature for 1 h and then with antibodies against either H3K9-Ac or mouse IgG (1 µl per IP; Active Motif) at 4 °C overnight. Next, 25 µl of pre-blocked agarose beads was added to each sample tube and rocked at 4 °C for 3 h. IP samples were washed sequentially with 0.1% SDS IP buffers with low and high sodium chloride concentrations, respectively. Reverse cross-linking was performed on all samples at 65 °C overnight. ChIP DNA was extracted by phenol/chloroform/isoamyl alcohol (pH 7-8, Fisher Scientific) at a ratio of 1:1 (v/v) and precipitated by incubating at −80 °C for 1 h after sequentially adding 3 M NaOAc (1:1, v/v, Fisher Scientific), glycogen (1 µl of 20 mg/ml stock; Roche), and 100% ethanol (1:2.5, v/v). The DNA pellets were washed with 500 µl of 70% ethanol and pelleted by centrifugation at 14,000×*g* at 4 °C for 5 min. The DNA pellets were air-dried, reconstituted in 30 µl of nuclease-Free water (Ambion, AM9938), and analyzed by qPCR (2 µl per reaction) with iTaq Universal SYBR Green Supermix (Bio-Rad, 1725122) on an iCycler iQ Real-Time PCR Detection System (Bio-Rad).

### Virus production and transduction

DNA encoding shRNA against *ASNS* in a pLKO.1 lentiviral backbone was obtained from the RNAi Consortium (Broad Institute). pΔ8.7 and pVSV-G were co-transfected with pLKO.1 using Lipofectamine 2000 (Life Technologies, 11668-019) into HEK293T cells to generate lentivirus, as described previously^3,6^. For viral transduction, the cells were infected with media containing viruses in the presence of polybrene (8.3 µg/mL), followed by puromycin (2 µg/mL) selection.

### Cell viability assay and siRNAs

The acid phosphatase (ACP) assay was used to measure cell viability, as described previously^3,6^. The cells (5000/well) were seeded into 96 well-plates and were incubated with various treatments as specified for 3 days. TSC1 (sc-37437), TSC2 (sc-36762), ATF3 (sc-29757), ATF4 (sc-35112), GCN2 (sc-45644) siRNAs were purchased from Santa Cruz. Each siRNA product is a pool of 3 target-specific siRNAs of 19–25 nt. Individual ASNS, ATF4, and XBP1 ON-TARGETplus siRNAs were acquired from Dharmacon (Supplementary Table 2). siRNA transfection was performed according to the protocol of the manufacturer. Cells were re-plated at 24 h post-transfection onto a 96-well plate and incubated in arginine-depleted medium for 72 h once they attached. ACP assay was conducted at the end-point of the experiment.

### Western blot and antibodies

Western blotting was conducted as described previously^3,6^. Immunoblots were visualized using the ChemiDoc Imaging System (Bio-Rad). After chemiluminescent reaction, blots were visualized with a ChemiDoc Touch Imaging System (Bio-Rad). The signal intensities of the captured images were analyzed with Image Lab Software (Bio-Rad, version 5.2.1). Results of Western analyses shown are representatives of at least three independent experiments. Antibodies used were OXPHOS Cocktail (MS501) from MitoSciences (1:1000); β-actin (MAB1501R) from Millipore (1:5000); GAPDH (sc-25778) from Santa Cruz (1:5000); H3 (39763; 1:10,000), H3K9me3 (61013; 1:1000), and H3K9Ac (61251; 1:1000) from Active Motif; eIF2α (2103S), p-eIF2α (9721S), p70S6K (9202), phospho-p70S6K (Thr389, 9234) and ATF4 (11815S) from Cell Signaling (1:1000 for all); Pan Acetyl-H3 (ab47915; 1:1000) from Abcam; HIF-1α (610958; 1:3000) from BD Biosciences; and ASNS (14681-1-AP; 1:3000) from Proteintech. The anti-ASS1 antibody was a gift from L.-J. Shen^6^.

### Intracellular ROS measurement

DCFDA (Sigma-Aldrich, D6883) was used for ROS measurement, as described previously^6^. Briefly, the cells were stained with DCFDA (10 nM) for 30 min. The oxidized DCF has a maximum emission at 530 nm and was analyzed using flow cytometry (Accuri C6, BD).

### Bioinformatics

The results herein are based in part upon data generated by The Cancer Genome Atlas (TCGA) Research Network (http://cancergenome.nih.gov/). TCGA Pan-Cancer RNA-Seq expression data (level 3 data from Illumina HiSeq platform from February 2015)^28^ were applied to the analysis of *ASS1* gene expression pattern. TCGA cancer types having no more than 5 samples of normal tissues were excluded from the analysis. Log2-transformed expression values were used for plotting and statistical analysis. Standard box plots were used to visualize the expression distribution and differences among different sample types. Statistical significance of the expression differences between groups was determined using Welch’s t-test.

### Oxygen consumption rate and extracellular acidification rate

Mitochondrial OCR and ECAR were measured using the Seahorse Bioscience XF96 or XF24 Extracellular Flux Analyzer (Agilent). Briefly, 2–8 × 10^4^ cells were seeded in cell culture microplates overnight. The next day, the medium was changed to arginine-deficient medium or media with various drug treatments. On the day of the experiment, the cells were washed and incubated with XF Assay medium (Agilent, 102365-100) supplemented with glucose (25 mM) and sodium pyruvate (1 mM) for OCR, or XF Base Medium (Agilent, 102353-100) supplemented with glutamine (2 mM) for ECAR. The pH value of the assay medium was adjusted to 7.4. OCR was measured following sequential injections of oligomycin (1 mM), FCCP (0.5 mM), and rotenone (2.5 mM), and ECAR was measured with sequential injections of glucose (80 mM), oligomycin (9 µM), and 2-deoxyglucose (1 M) according to the manufacturer’s instructions. The OCR and ECAR measurements were normalized to cell numbers.

### Glutamate-stimulated NADH autofluorescence assay

The cells were trypsinized and maintained in a resting condition, i.e., low glucose Krebs-Ringer bicarbonate-HEPES buffer medium containing NaCl (135 mM), KCl (3.6 mM), HEPES (10 mM), NaHCO_3_ (5 mM), NaH_2_PO4 (0.5 mM), MgCl_2_ (0.5 mM), CaCl_2_ (1.5 mM) and glucose (0.5 mM). Then, cells were stimulated by glutamate (200 mM) in the dark for 15 min. Autofluorescence of NADH was measured using a BD LSRFortessa cell analyzer (BD Biosciences) with excitation and emission filters set at 350 nm and 490 nm, respectively. The experimental values were corrected by subtracting the background.

### Xenograft mouse model, arginine-free diet, and tumor characterization

Animal experiments were approved by the Institutional Animal Care and Use Committee at City of Hope. Luciferase-tagged BT-549 cells (3.3 × 10^5^) were injected into the mammary fat pads of 6-week-old female *NOD.Cg-PrkdcscidIl2rgtm1Wjl/SzJ* (NSG) mice. The mice were separated into 2 groups with 8 mice in each group. One group was fed with a control diet (Teklad, TD.01804) and the second group was fed with an arginine-free diet (Teklad, TD.09152). The diet was initiated one week prior to tumor inoculation. Tumor size and mouse weight were measured twice a week. Tumor size was calculated with the equation (LxW^2^)/2, where L is length and W is width of the tumor. The tumor masses were excised *en bloc* and fixed in 10% neutral-buffered formalin for 48 h. Tissue embedding, sectioning, and staining with hematoxylin and eosin were performed in the City of Hope Pathology Core. Briefly, after deparaffinization and rehydration, the sections were stained with hematoxylin for 4 min, rinsed in tap water, and destained with 0.3% acid alcohol. After rinsing in tap water, the sections were stained with eosin for 2 min, followed by dehydration, clearing, and mounting. The mitotic cells were enumerated in 5 non-overlapping 5× or 10× fields and counted with Image-Pro Premier 9.0 (Media Cybernetics); slides from at least 3 tumors were included in each group.

### In vivo bioluminescence imaging

For four weeks after tumor cell inoculation, the mice were imaged every week with Ami HTX optical imaging system (Spectral Instruments Imaging). Before each imaging session, the mice were injected with D-luciferin (150 mg/Kg in saline, PerkinElmer, 122799) and exposed to a 2–5% isoflurane/oxygen mixture for anesthesia. Images were taken 8 min after injection of D-luciferin and with 20 s and 40 s exposure times.

### Statistical analysis

Data with error bars are presented as mean ± S.D. Student’s two-tailed *t* test was used to determine the *p*-value. Differences were considered statistically significant when the *p*-value was <0.05.

## Electronic supplementary material

Supplementary Information

## Data Availability

RNA sequencing data shown in Figs. 2a and 4a are deposited at Gene Expression Omnibus (GEO) database of NCBI. The data set can be accessed by its accession code GSE104105. Metabolomics data presented in Fig. 1 is deposited at MetaboLights of EMBL-EBI. The complete dataset can be accessed with the identifier MTBLS745. All other relevant data present in this paper are available in this article or from the authors upon request.
