# Supplementary material for: Arginine starvation kills tumor cells through aspartate exhaustion and mitochondrial dysfunction
Source: Commun Biol. 2018 Oct 26;1:178. doi: 10.1038/s42003-018-0178-4 (PMC6203837; doi:10.1038/s42003-018-0178-4)
Supplement: Supplementary file 1 — Supplementary Information [file 42003_2018_178_MOESM1_ESM.pdf]

Supplementary Figure 1

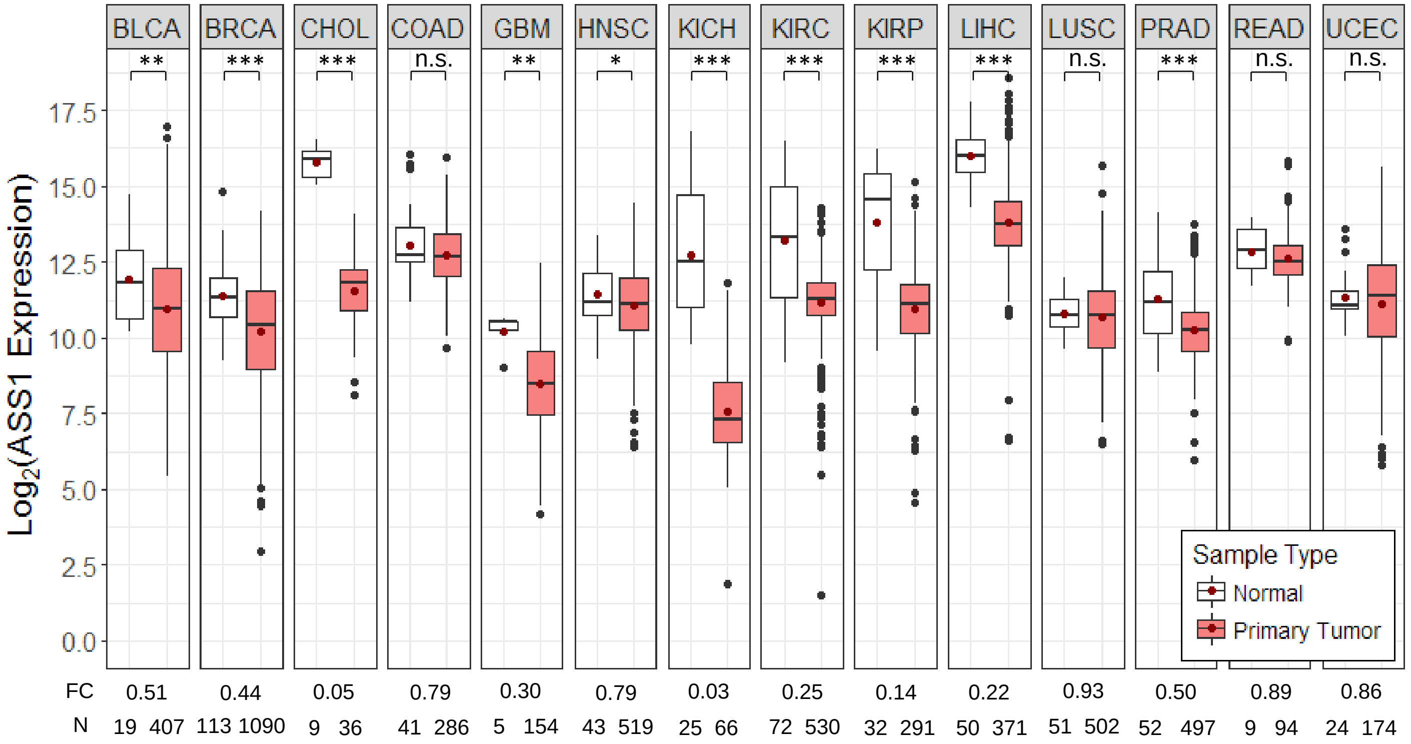

Supplementary Figure 1. Down-regulation of *ASS1* gene expression across multiple human cancer types. Data from The Cancer Genome Atlas (TCGA) Pan-Cancer project were applied to compare the expression levels between primary tumors and normal tissues using RNA-Seq data. Sixteen cancer types, having at least 5 samples for both normal tissues and primary tumors, were included in this analysis. Red dots in the standard box-plots indicate the average expression values. Fold change (FC) of the normalized expression values and the number (N) of samples are labeled at the bottom. BLCA: Bladder urothelial carcinoma, BRCA: Breast invasive carcinoma, CHOL: Cholangiocarcinoma, COAD: Colon adenocarcinoma, GBM: Glioblastoma multiforme, HNSC: Head and neck squamous cell carcinoma, KICH: Kidney chromophobe, KIRC: Kidney renal clear cell carcinoma, KIRP: Kidney renal papillary cell carcinoma, LIHC: Liver hepatocellular carcinoma, LUSC: Lung squamous cell carcinoma, PRAD: Prostate adenocarcinoma, READ: Rectum adenocarcinoma, and UCEC: Uterine corpus endometrial carcinoma. *p*-values between groups were determined using Welch's t-test. n.s., not significant; *: $p < 0.05$; **: $p < 0.01$; ***: $p < 0.001$.

Supplementary Figure 2

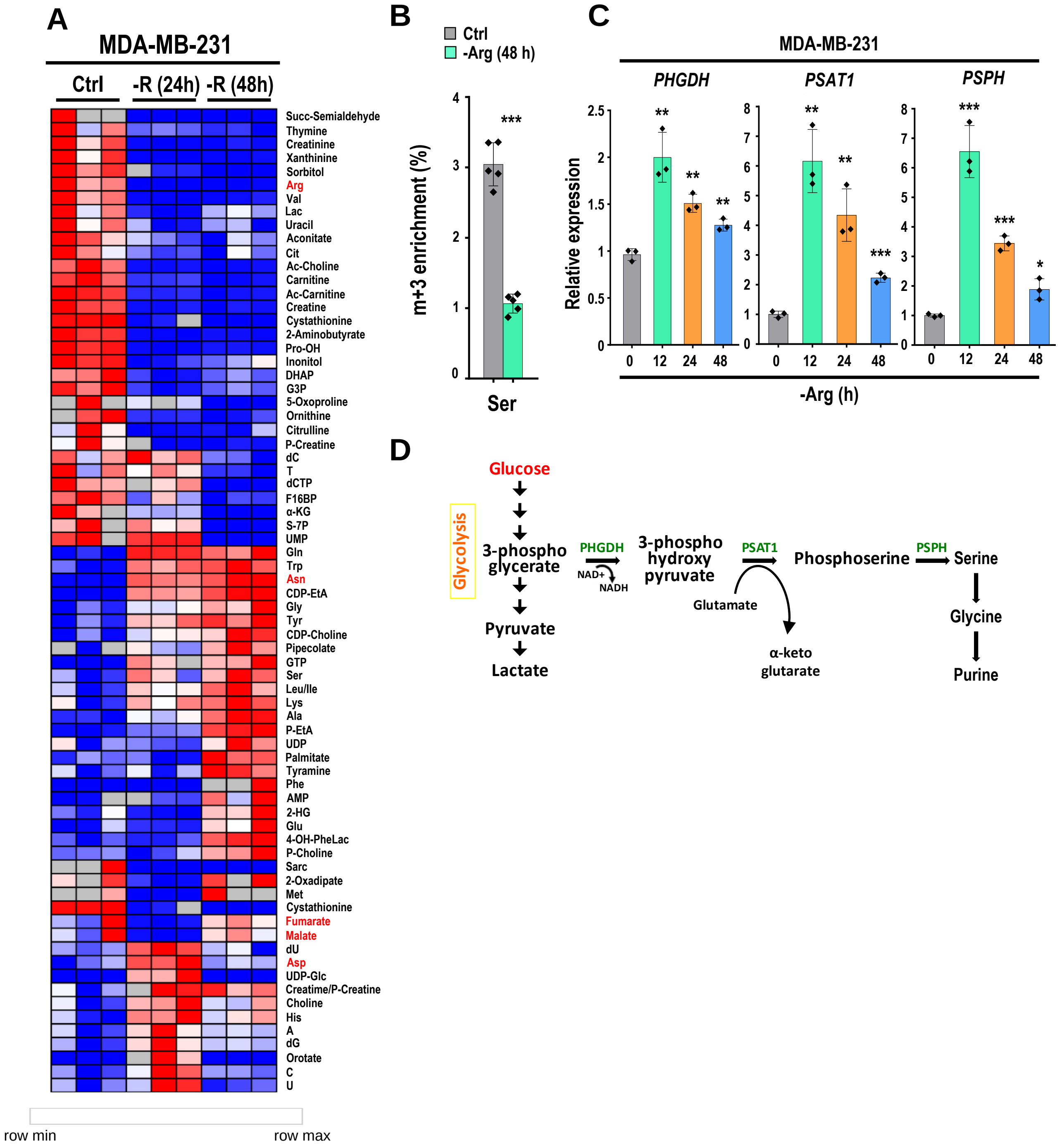

Supplementary Figure 2. Alterations in glycolysis and TCA intermediates after arginine starvation. (A) Clustering analysis and heatmap of metabolites from MDA-MB-231 cells cultured in full (Ctrl) or arginine-starved (-R; 24 and 48 h) medium (n=3 per group). Data are shown on the log2 scale. Notable changes in arginine (Arg), asparagine (Asn), aspartate (Asp), fumarate (Fum), malate (Mal) are highlighted in red. **(B)** [^{13}C] m+3 enrichment of serine from [U-^{13}C] glucose in MDA-MB-231 cells under 48 h arginine starvation; n=5. **(C)** qRT-PCR analyses of *PHGDH, PSAT1* and *PSPH* expression during arginine starvation in MDA-MB-231 cells. For bar graphs, data are shown as mean ± S.D.; n=3. *: $p < 0.05$; **: $p < 0.01$. **(D)** Diagram of directing glycolytic intermediate to serine/glycine biosynthesis.

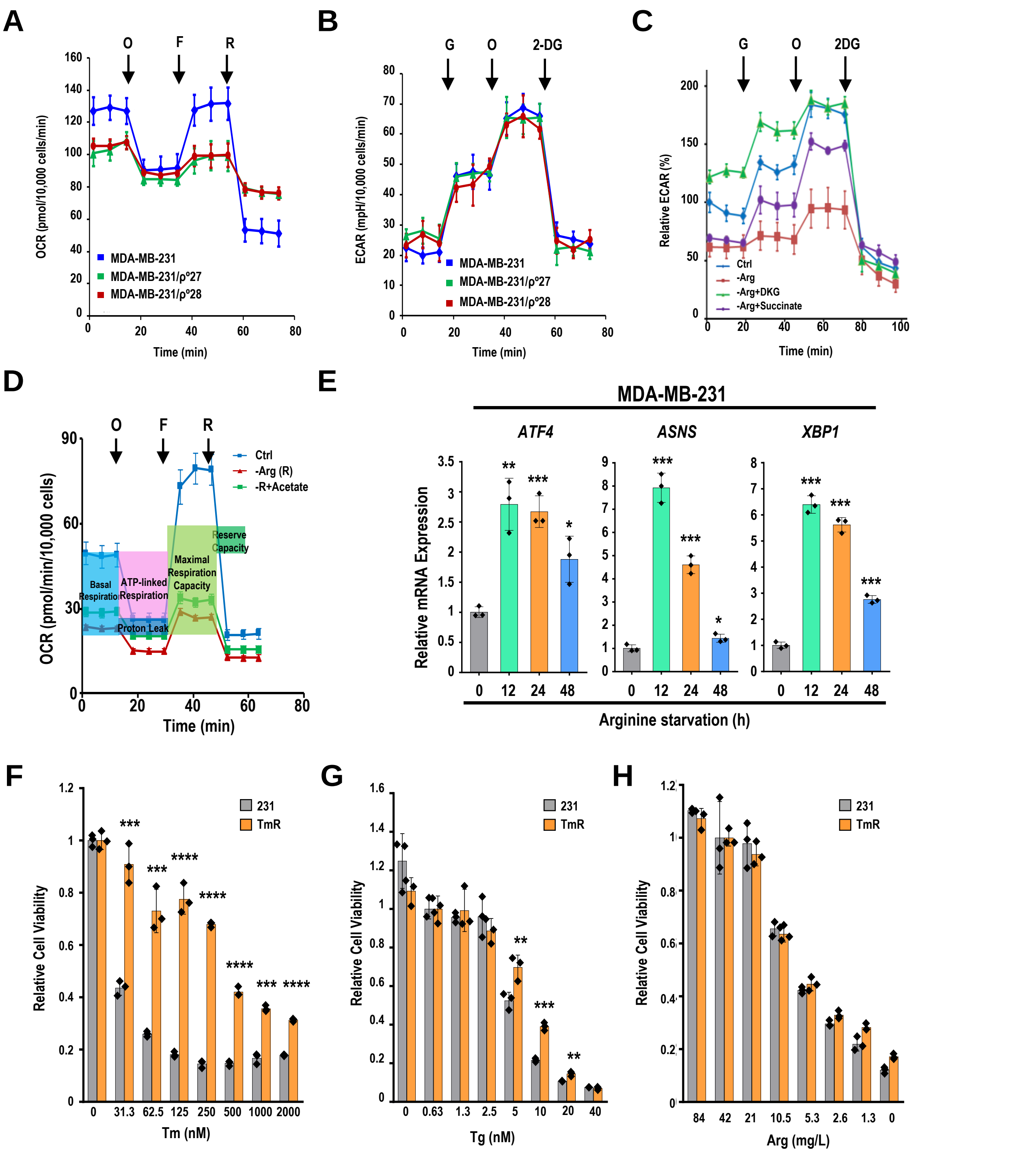

Supplementary Figure 3. Mitochondria and *ASNS* are targets of arginine starvation. (A) The oxygen consumption rate (OCR) and **(B)** extracellular acidification rate (ECAR) were measured in cells lacking mitochondria (ρ^0 cells); n=5. **(C)** ECAR of arginine-starved MDA-MB-231 cells supplemented with succinate (10 mM) or the α-ketoglutarate analogue dimethyl-2-ketoglutarate (DKG, 10 mM); n=5. Relative ECAR was calculated by designating the basal ECAR of cells in full medium as 100%. **(D)** Effect of arginine starvation and replenishment with acetate (10 mM) on OCR measured using a Seahorse bioanalyzer in MDA-MB-231 cells; n=5. **(E)** qRT-PCR analyses of *ATF4, ASNS* and *XBP1* mRNA expression in MDA-MB-231 cells at different time points of arginine starvation; n=3. **(F-H)** Cell viability of tunicamycin-resistant (TmR) MDA-MB-231 cells in response to **(F)** tunicamycin (Tm) or **(G)** thapsigargin (Tg) treatment. **(H)** Comparison of cell viability after arginine starvation for TmR and parental cells. **(A-D)** O: oligomycin; F: FCCP; R: rotenone; G: glucose; 2DG: 2-deoxyglucose. For bar graphs, data are shown as mean ± S.D.; n=3. **: $p < 0.01$; ***: $p < 0.001$; ****: $p < 0.0001$.

Supplementary Figure 4

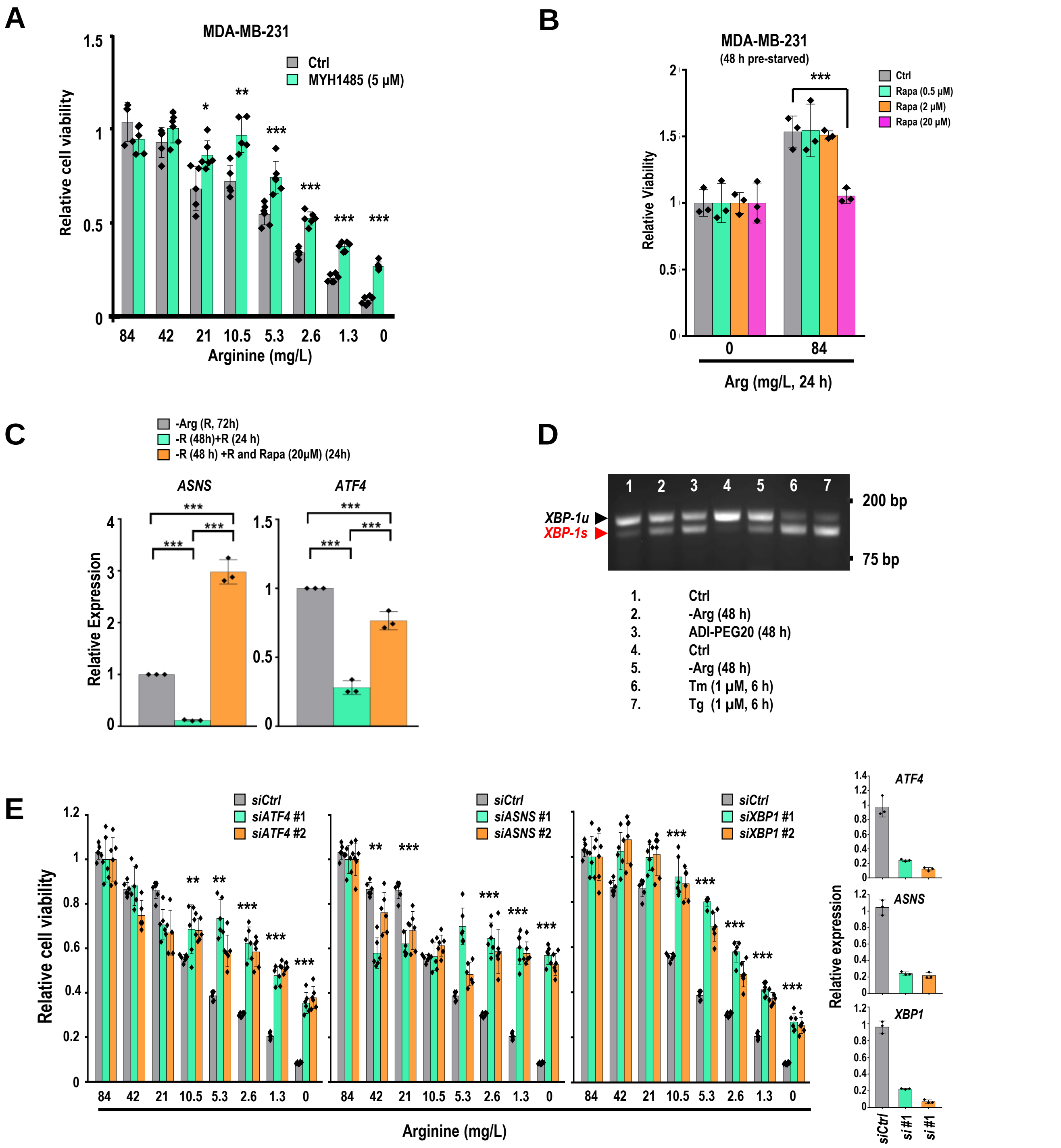

Supplementary Figure 4. Figure S4. Arginine starvation-induced ATF4-ASNS signaling is partially mediated through mTOR pathway. (A) Effect of mTOR activator MHY1485 (5μM) on cell viability of MDA-MB-231 cells upon arginine starvation; n=5. **(B)** Effect of rapamycin on cell viability rescue (24h) after 48 h arginine starvation. MDA-MB-231 cells were first starved with arginine free medium for 48 h and replenished with arginine free, full medium or full medium containing different concentrations of rapamycin for 24 h; n=3. **(C)** qRT-PCR measurements of *ASNS* and *ATF4* mRNA level in arginine starved MDA-MB-231 cells after 24 h rescue with or without rapamycin (20 μM, 24 h); n=3. **(D)** *XBP1* mRNA alternative splicing was analyzed by RT-PCR using total RNA extracted from arginine starved MDA-MB-231 (lane1-3,6,7) and BT549 cells (lane 4,5). Tm: tunicamycin; Tg: thapsigargin; *XBP-1u*: un-spliced *XBP-1*; *XBP-1s*: spliced *XBP-1*. **(E)** Cell viability of MDA-MB-231 cells under arginine starvation with ATF4, ASNS, or XBP1 down-regulation by siRNAs. Knockdown of *ATF4*, *ASNS*, and *XBP1* were confirmed by qRT-PCR; n=3. Data are shown as mean ± S.D.; n=5. *: $p < 0.05$; **: $p < 0.01$; ***: $p < 0.001$.

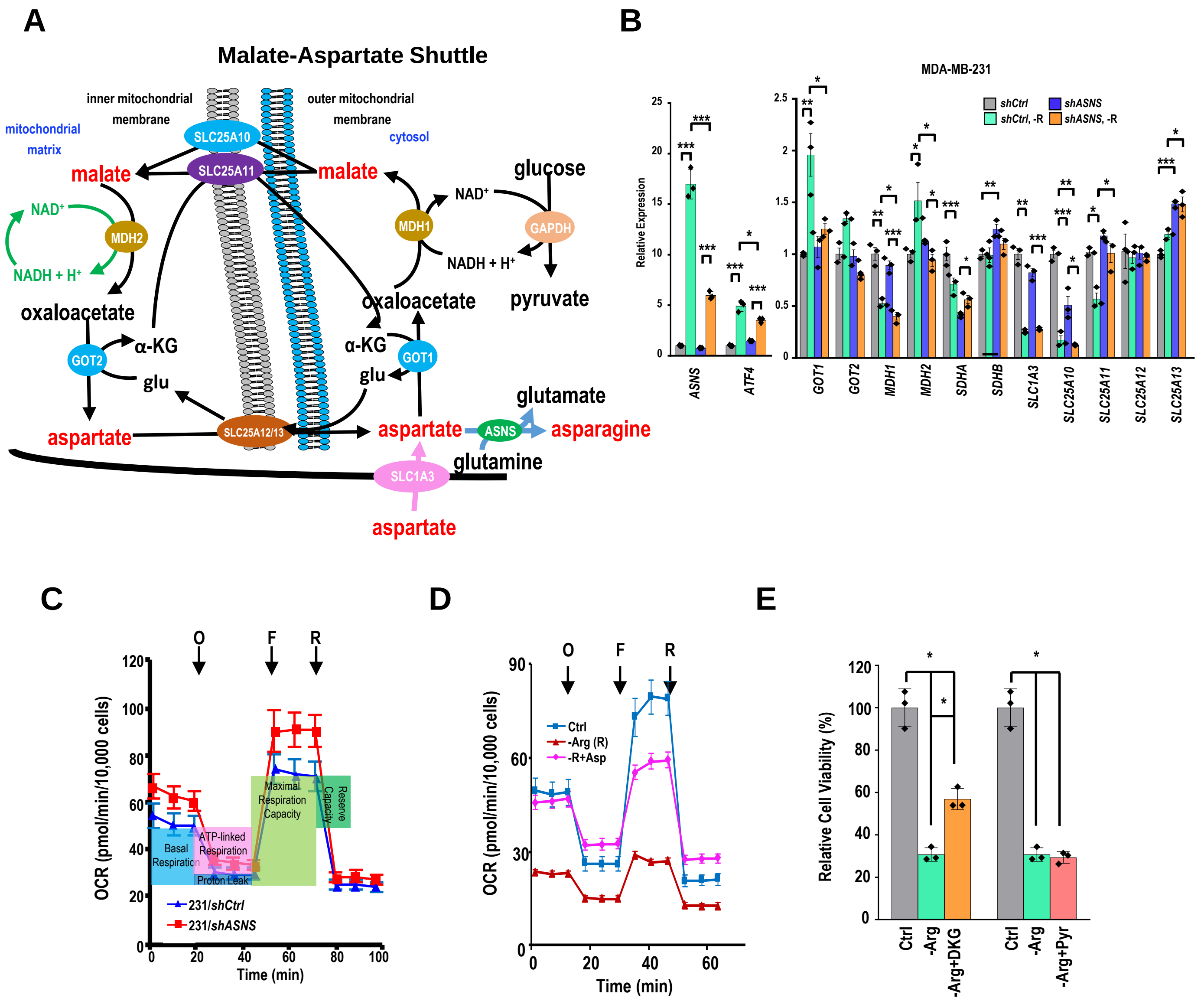

Supplementary Figure 5. Arginine starvation suppresses the malate-aspartate shuttle. (A) Schematic of the malate-aspartate shuttle and ASNS. The malate-aspartate shuttle translocates electrons produced during glycolysis across the mitochondrial membrane to fuel OxPhos. Specifically, cytosolic malate dehydrogenase 1 (MDH1) converts oxaloacetate and NADH to malate and NAD^+. Once malate is formed, SLC25A10 and SLC25A11 import the malate from the cytosol into the mitochondrial matrix[1], where malate (and NAD^+) is converted back to oxaloacetate (and NADH) by mitochondrial malate dehydrogenase 2 (MDH2). Subsequently, mitochondrial aspartate aminotransferase 2 (GOT2) converts oxaloacetate to aspartate, which is exported to cytoplasm by SLC25A12 and SLC25A13[1]. α-KG: α-ketoglutarate; glu: glutamate. **(B)** qRT-PCR analyses of *ASNS*, *ATF4* and malate-aspartate shuttle gene expression in MDA-MB-231 cells with or without arginine, and in *ASNS*-knockdown cells. The relative mRNA abundance was calculated by designating the mRNA abundance in *shCtrl*-cells as 1. **(C)** OCR was measured in *shCtrl*- and *shASNS*-cells. Data are shown as mean ± S.D.; n=8. **(D)** Effect of arginine starvation (-R; 48h) and replenishment with aspartate (Asp, 10 mM) on OCR in MDA-MB-231 cells; n=3. **(C, D)** O: oligomycin; F: FCCP; R: rotenone. (**E**) Cell viability of pyruvate (10 mM)- or dimethyl-α-ketoglutarate (DKG, 10 mM)-supplemented arginine-starved MDA-MB-231 cells. For bar graphs, data are shown as mean ± S.D.; n=3. *: $p < 0.05$; **: $p < 0.01$; ***: $p < 0.001$.

Supplementary Figure 6

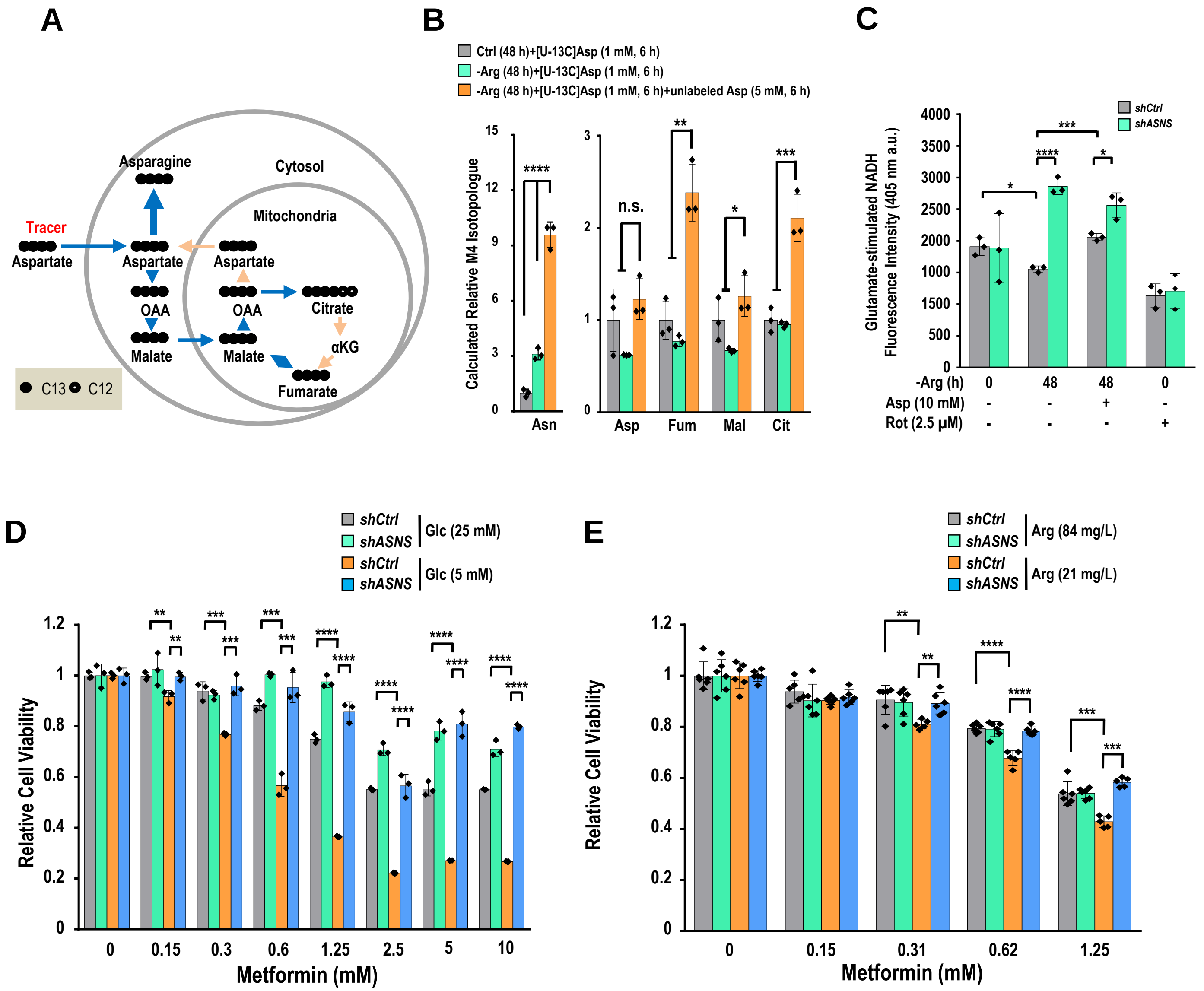

Supplementary Figure 6. Aspartate supplementation rescues the production of TCA intermediates, NAD$^+$/NADH ratio and NADH abundance in arginine-starved MDA-MB-231 cells. **(A)** Illustration of [U-^{13}C]aspartate tracing. For simplicity, only the major first round reactions of aspartate catabolism are shown in blue lines. Open circles represent natural carbon 12, and the solid circles indicate the carbon 13 isotope. OAA: oxaloacetate; αKG: α-ketoglutarate. **(B)** Control (+Arg) or arginine starved (-Arg, 48 h) MDA-MB-231 cells were cultured with [U-^{13}C]aspartate (1 mM) for 6 h. The relative aspartate-derived m+4 fractions of intracellular asparagine (Asn), aspartate (Asp), fumarate (Fum), malate (Mal), and citrate (Cit) were measured with GC mass spectrometry. As [U-^{13}C]aspartate only contributes to 1/6 of the total aspartate in the 6 mM condition, the data are shown as 6x the original measurement ; n=3. **(C)** Glutamate-stimulated NADH levels in *shCtrl*- and *shASNS*-MDA-MB-231 cells after arginine starvation with or without aspartate (Asp) or rotenone (Rot, control) measured by autofluorescence; n=3. **(D)** Viability of *shCtrl*- and *shASNS*-MDA-MB-231 cells in response to metformin treatment and glucose modulation ; n=3. **(E)** Viability of *shCtrl*- and *shASNS*-MDA-MB-231 cells in response to metformin treatment and arginine modulation. Cells were incubated with various concentrations of metformin in low glucose (5 mM) medium supplied with either high (84 mg/L) or low (21 mg/L) arginine for 72 h ; n=6.

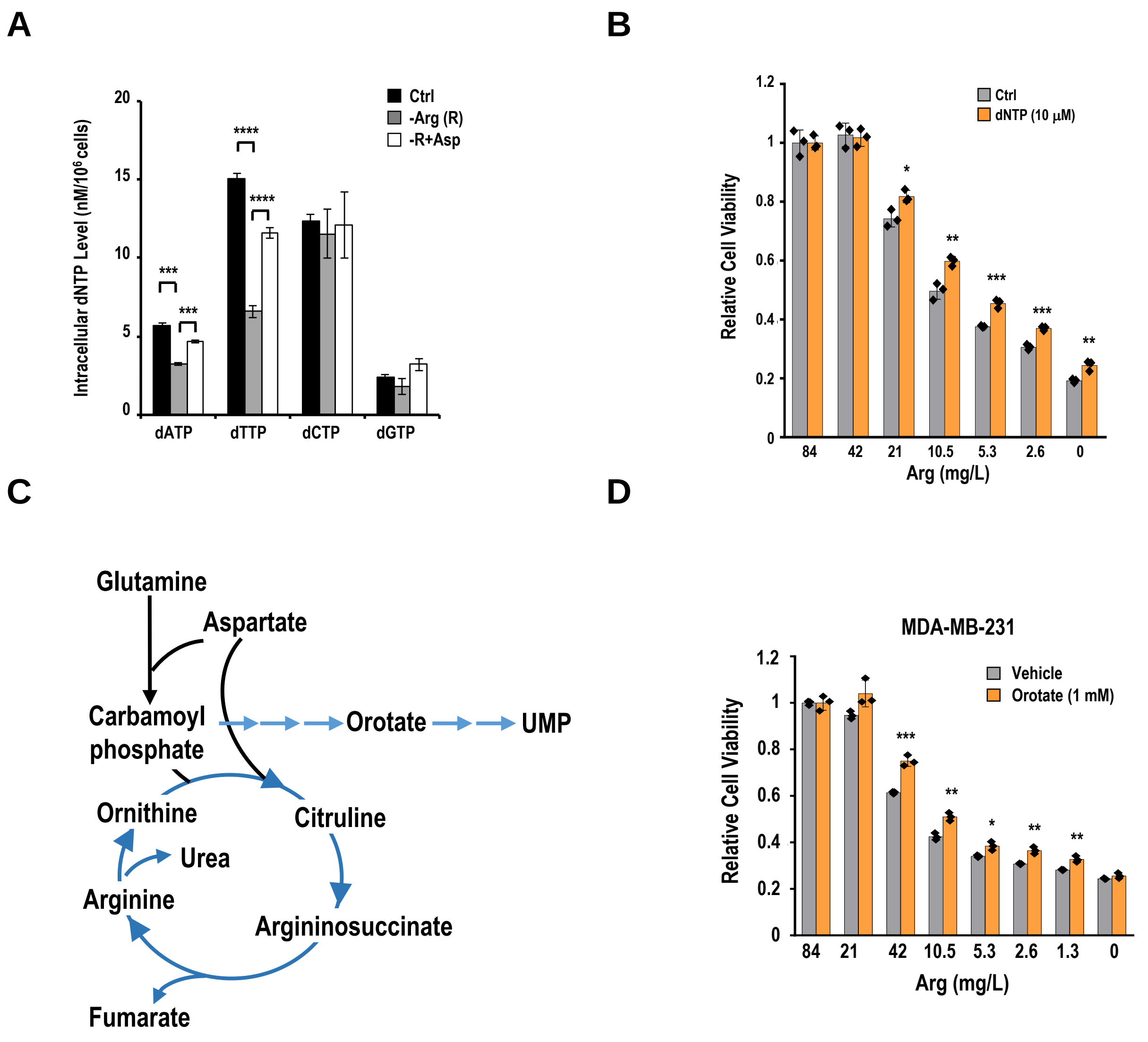

Supplementary Figure 7. Arginine Starvation Impairs Nucleotide Biosynthesis. (A) Aspartate rescues dATP and dTTP pools after arginine deprivation in MDA-MB-231 cells. Aspartate (Asp, D; 10 mM) was supplemented to full (Ctrl) or arginine-free (-Arg, -R) medium for 48 h. (**B**) Supplementation with dNTP (10 µM) rescues cell viability upon arginine depletion; n=3. Cell viability was measured by ACP assay. (**C**) Schematic of the *de novo* pyrimidine nucleotide synthesis pathway and its coupling with urea cycle. (**D**) MDA-MB-231 ells were incubated with decreasing concentrations of arginine in the absence or presence of orotate (1 mM) for 72 h. For bar graphs, data are shown as mean ± S.D.; n=3; *: $p < 0.05$; **: $p < 0.01$; ***: $p < 0.001$; ****: $p < 0.0001$.

Supplementary Figure 8

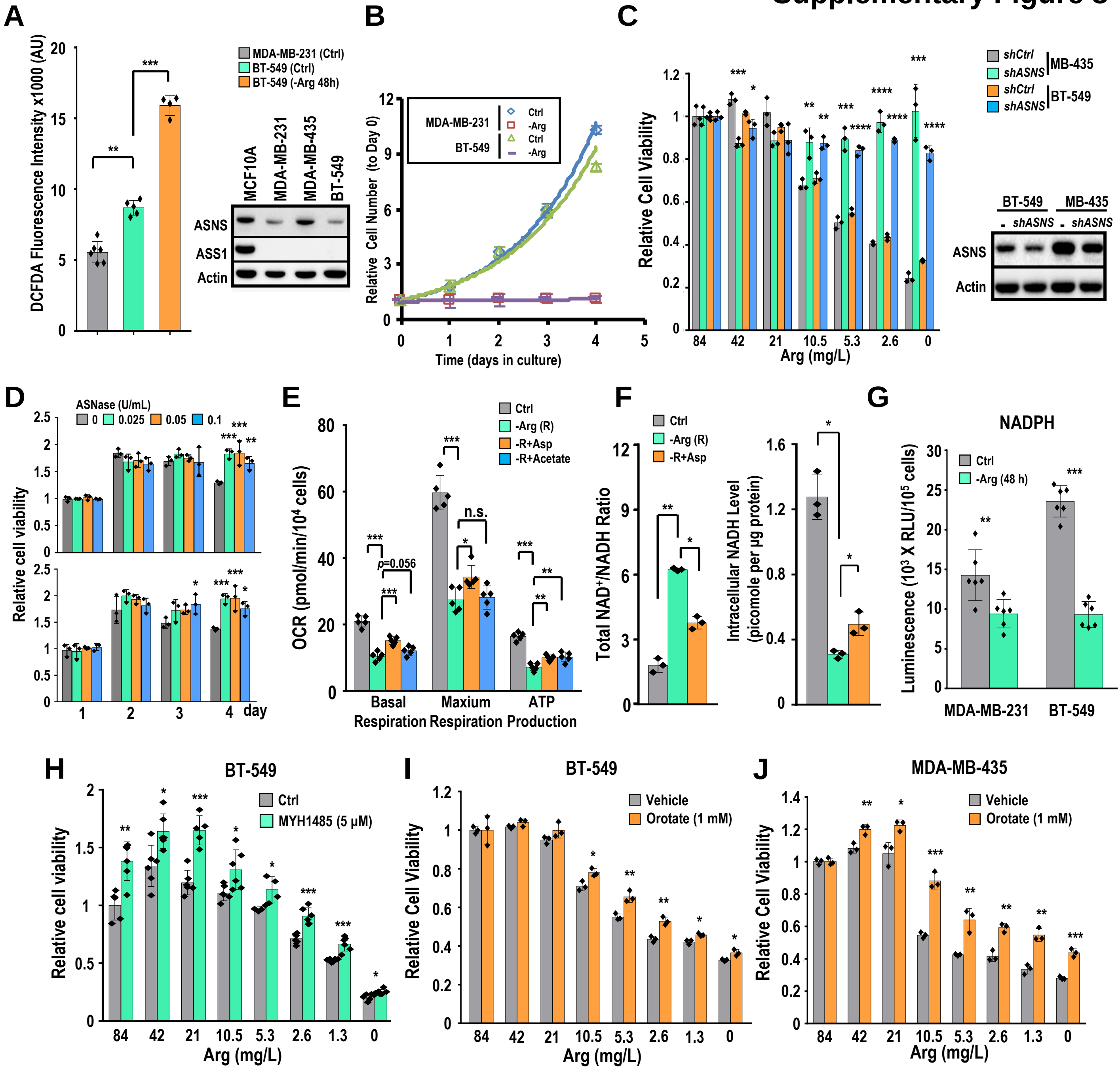

Supplementary Figure 8. Arginine starvation alters metabolism in ASS1-low breast cancer BT-549 cells. (A) ROS production was measured by DCF oxidation with or without arginine (-Arg) (*left panel*). Representative Western blots show the ASNS and ASS1 levels in breast cancer MDA-MB-231, MDA-MB-435 and BT-549 cells and immortalized breast MCF10A cells (*right panel*) ; n=5. **(B)** Growth curve of MDA-MB-231 and BT-549 cells cultured in full (Ctrl) or arginine-depleted (-Arg) media. The relative cell number was determined using trypan blue exclusion cell counting and normalized to the number of seeded cells. **(C)** Cell viability in arginine-starved, *ASNS*-knockdown MDA-MB-435 and BT-549 cells (*left panel*). Representative Western blots confirm *ASNS* knockdown in MDA-MB-435 and BT-549 cells (*right panel*); n=3. **(D)** Cell viability of BT-549 (*upper* panel) and MDA-MB-231 cells (*lower* panel) in the presence of indicated concentrations of ASNase and asparagine (8mM) during the course of arginine deprivation. Cell viability was measured every 24 h for 4 consecutive days. The relative cell viability was calculated by designating the day 1 ACP reading of cells in respective conditions as 1; n=3. **(E)** Effect of arginine starvation and replenishment with aspartate (10 mM) or acetate (10 mM) on basal respiration, maximal respiration, and ATP production in BT-549 cells ; n=5. **(F)** Replenishment with aspartate (10 mM) rescues the NAD^+/NADH ratio and NADH abundance in arginine-starved BT-549 cells. **(G)** Intracellular NADPH level in MDA-MB-231 and BT-549 cells after 48 h arginine starvation, n=6. (**H**) Effect of mTOR activator MHY1485 on the survival of BT-549 cells under arginine starvation ; n=4. (**I, J**) Cells were incubated with decreasing concentrations of arginine in the absence or presence of orotate (1 mM) for 72 h in BT-549 (**I**) and MDA-MB-435 (**J**). For bar graphs, data are shown as mean ± S.D.; n=3; *: $p < 0.05$; **: $p < 0.01$; ***: $p < 0.001$; ****: $p < 0.0001$.

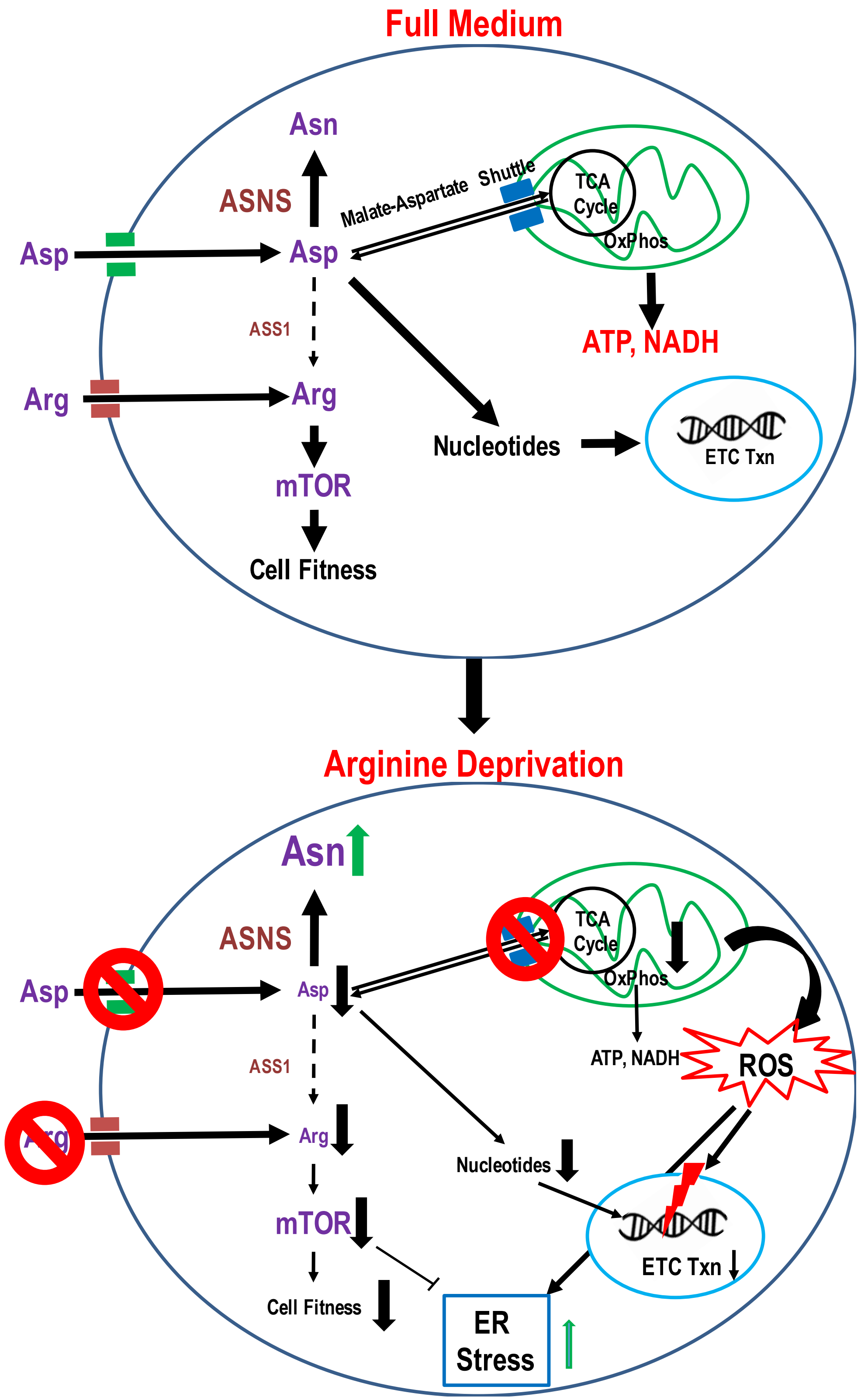

Supplementary Figure 9. Schematic model illustrates the relationship among arginine starvation, mitochondrial damage, and cell death. Arginine starvation inhibits mTOR, induces the upregulation of ASNS, which converts aspartate to asparagine, and down-regulates the malate-aspartate shuttle in arginine auxotrophic (ASS1-deficient) cancer cells. Arginine starvation also silences nuclear-encoded mitochondrial genes in a coordinated way, impairing mitochondrial function. The combined effect of decreased aspartate and impaired mitochondrial function leads to a depleted nucleotide reduction, non-canonical ER stress, and increased ROS production, resulting in consequent DNA damage and cell death.

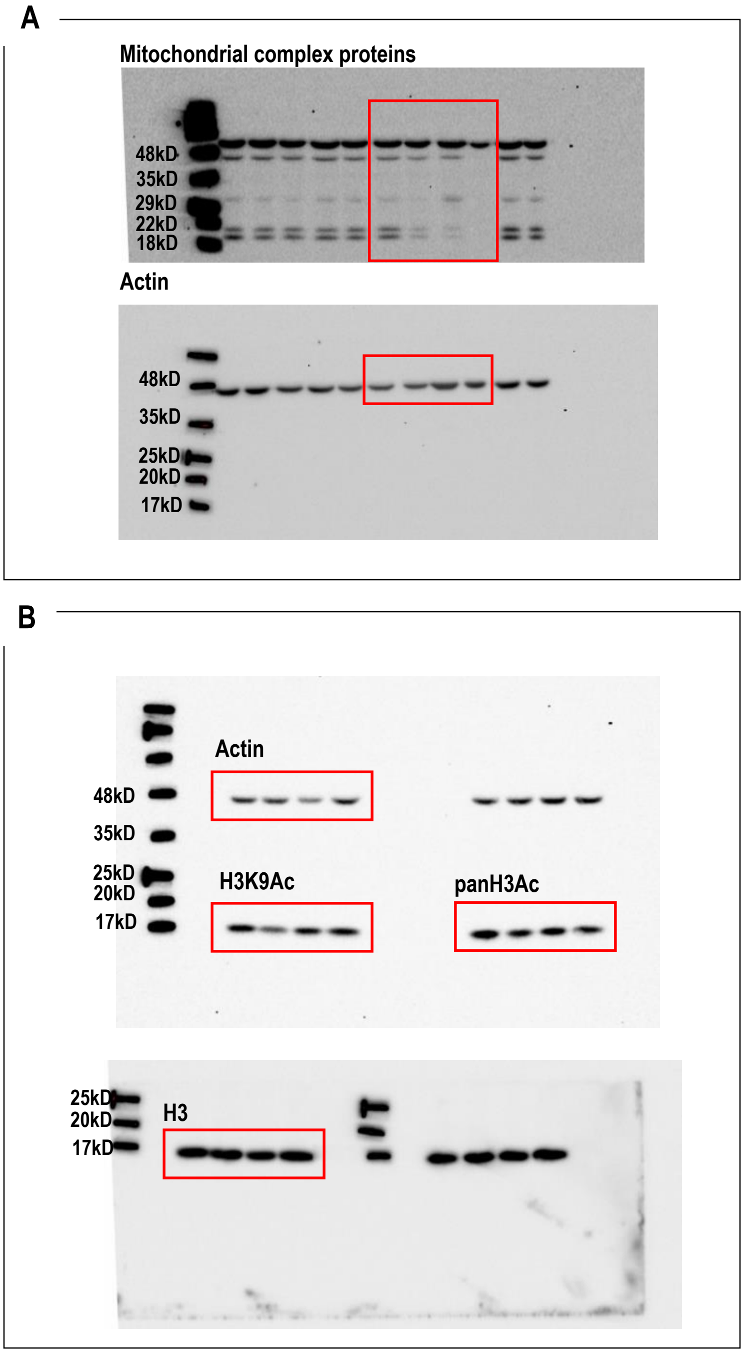

Supplementary Figure 10. Uncropped blots.
(**A**) Uncropped Western blots illustrating molecular weight markers of Fig. 2B. (**B**) Uncropped Western blots with molecular weight markers of Fig. 3B.

Supplementary Figure 11

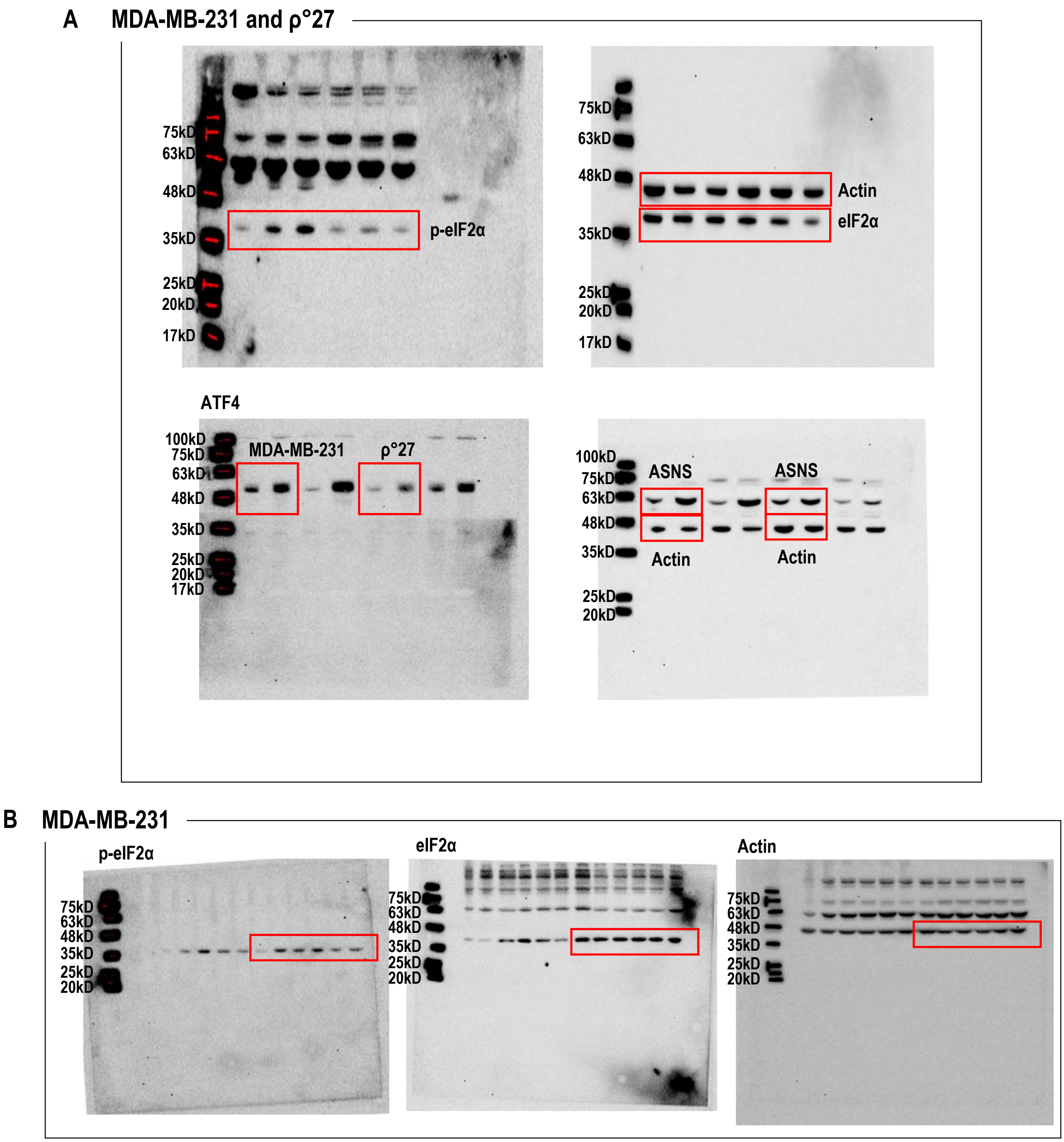

Supplementary Figure 11. Uncropped blots. (**A**) Uncropped Western blots illustrating molecular weight markers of Fig. 4B upper and middle panels. (**B**) Uncropped Western blots with molecular weight markers of Fig. 4B lower panel.

Supplementary Figure 12

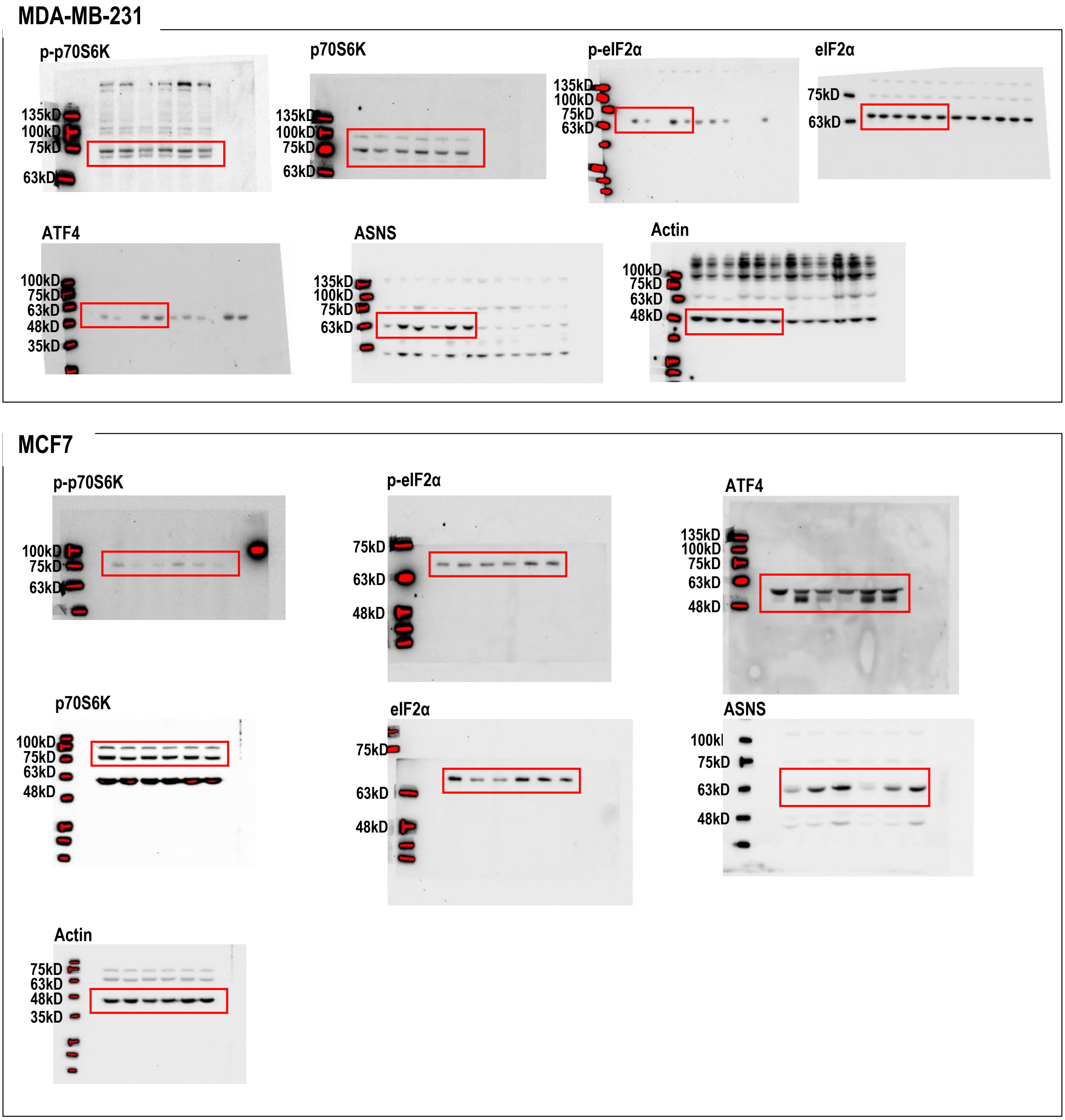

Supplementary Figure 12. Uncropped blots. Uncropped Western blots demonstrate molecular weight markers of Fig. 4C.

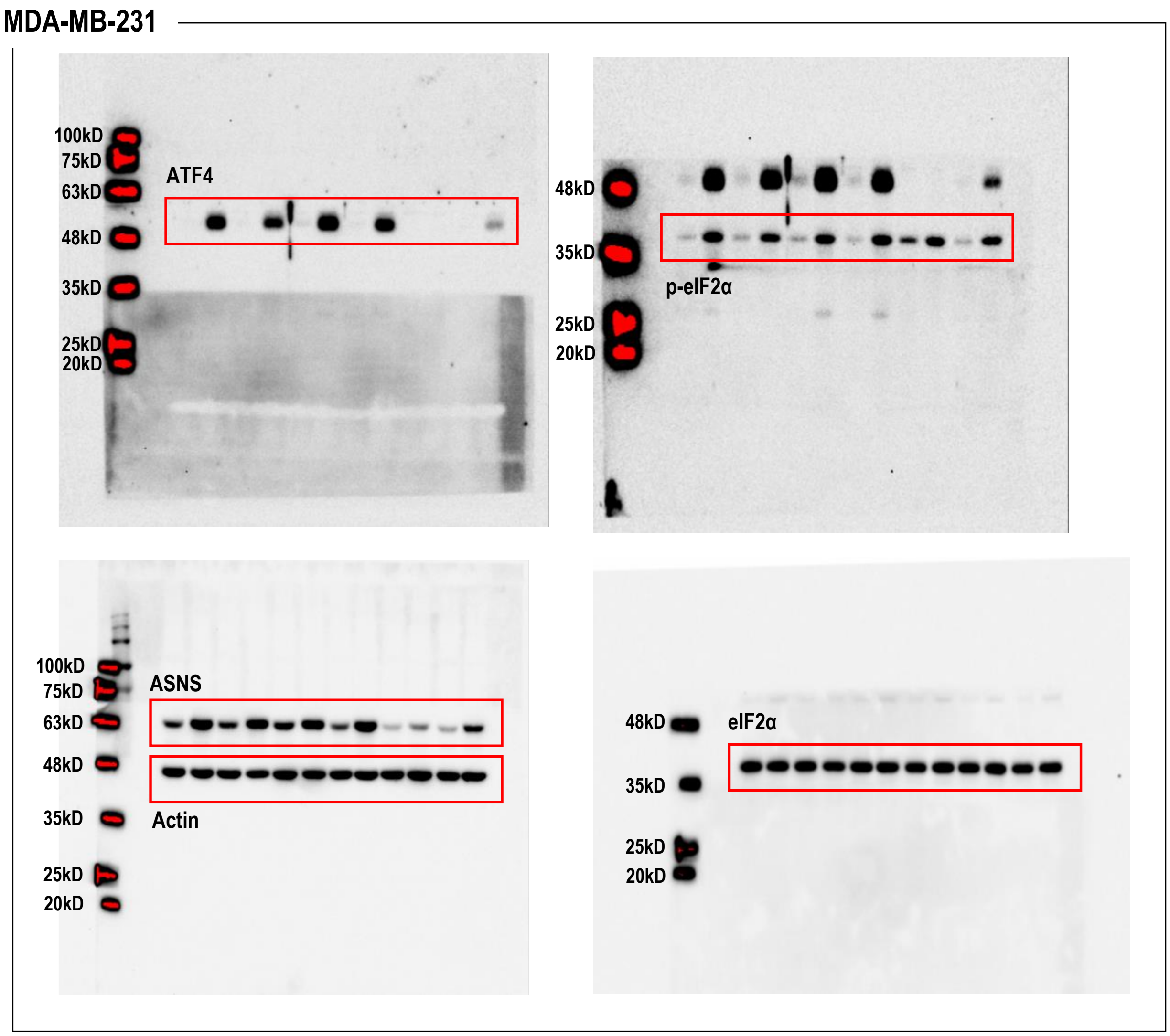

Supplementary Figure 13. Uncropped blots. Uncropped Western blots demonstrate molecular weight markers of Fig. 4D.

Supplementary Table 1. Primers used for RT-PCR

Gene	Forward	Reverse
NDUFA6	5'-TTCACTCATGTACTTGTACCTGTC-3'	5'-TCTGGACTTCTTTTCAACTATGCC-3'
NDUFA7	5'-CTGCTCAAGTGCAAGCGTGACA-3'	5'-CGCTCAAACTCCTTCATGCGCA-3'
NDUFB10	5'-CAGCCCGTTCTTGTATTCCG-3'	5'-ACTTCCTGGCAGAACTGTAGGC-3'
NDUFV1	5'-TGTGTGAGACGGTGCTGATGGA-3'	5'-CGATGGCTTTCACGATGTCCGT-3'
ATF4	5'-TTCTCCAGCGACAAGGCTAAGG-3'	5'-CTCCAACATCCAATCTGTCCCG-3'
ASNS	5'-AACAGAGTGGCAGCAACCAAGC-3'	5'-CTGTGAAGAACAACCTCAGGATC-3'
GOT1	5'-GGACCTGGAACCACATCACTGA-3'	5'-ACCACTTGGCAGCAGGTAGATG-3'
GOT2	5'-AGTGAAGGCTCCTACACGCTCA-3'	5'-CCAAGGCTTTGCCAGTGGTGAT-3'
MDH1	5'-CGGTGTCCTAATGGAACTGCAAG-3'	5'-CATCCAGGTCTTTGAAGGCAACG-3'
MDH2	5'-CTGGACATCGTCAGAGCCAACA-3'	5'-GGATGATGGTCTTCCCAGCATG-3'
SDHA	5'-GAGATGTGGTGTCTCGGTCCAT-3'	5'-GCTGTCTCTGAAATGCCAGGCA-3'
SDHB	5'-GCAGTCCATAGAAGAGCGTGAG-3'	5'-TGTCTCCGTTCCACCAGTAGCT-3'
SDHC	5'-GGTTCAAACCGTCCTCTGTCTC-3'	5'-CGACATGCCAAAAAGAGAGACCC-3'
SDHD	5'-GCAGCACATACACTTGTCACCG-3'	5'-GGGAATAGTCCATCGCAGAGCA-3'
SLC1A3	5'-GGTTGCTGCAAGCACTCATCAC-3'	5'-CACGCCATTGTTCTCTTCCAGG-3'
SLC25A10	5'-GCAGACTTGGTCAACGTCAGGA-3'	5'-CATGGTTGCACCCGAGAACAGT-3'
SLC25A11	5'-TCAGCGGTCTTGTCACCAC-3'	5'-CAGCCCGTTCTTGTATTCCG-3'
SLC25A12	5'-GGGCTTCTTTGGACTCTACAGG-3'	5'-GGAAGTGGAACAGAGCCATCTC-3'
SLC25A13	5'-AGATGGTTCGGTCCCACTTGCA-3'	5'-ACCAGTGGTGATTTCTCCTGCC-3'
TFAM	5'-GGGCGGCCGGGGACAGAG-3'	5'-TAGGAAGGGCAGGGAGTGAAGGAA-3'
XBP1	5'-CCTGGTTGCTGAAGAGGAGG-3'	5'- CCATGGGGAGATGTTCTGGAG-3'

Supplementary Table 2. Individual siRNAs used for gene down-regulation

Target genes	Targeting sequence	Catalog number
**Human** *ASNS*	5'-GGGUAGAGAUACAUAUGGA-3'	J009377-06
**Human** *ASNS*	5'-GGUGAAAUCUACAACCAUA-3'	J009377-08
**Human** *ATF4*	5'-CAGAUUGGAUGUUGGAGAA-3'	J005125-10
**Human** *ATF4*	5'-GAGAUAGGAAGCCAGACUA-3'	J005125-13
**Human** *XBP1*	5'-GAACAUCUCCCCAUGGAUU-3'	J009552-07
**Human** *XBP1*	5'-GGUAUUGACUCUUCAGAUU-3'	J009552-10

Supplementary Reference

1. Palmieri, F. The mitochondrial transporter family SLC25: identification, properties and physiopathology. *Mol Aspects Med* **34**, 465-84 (2013).
